# Tunable Fabrication of 2D‐Layered Magnesium Nanosilicates With Hemostatic Properties

**DOI:** 10.1002/smsc.70374

**Published:** 2026-09-09

**Authors:** Anna L. Keller, Kanwar Abhay Singh, Saptarshi Biswas, Kavita Kadu, Sarah E. Miller, Weijian Hua, Sridevi Conjeevaram, Vaaridhi Ramanuja, Shounak Roy, Samantha Foster, Changwoo Do, Wei‐Ren Chen, Yifei Jin, Akhilesh K. Gaharwar

**Affiliations:** ^1^ Department of Biomedical Engineering College of Engineering Texas A&M University College Station Texas USA; ^2^ Mechanical Engineering Department University of Nevada Reno Reno Nevada USA; ^3^ Interdisciplinary Program in Genetics Texas A&M University College Station Texas USA; ^4^ Neutron Scattering Division Oak Ridge National Laboratory Oak Ridge Tennessee USA; ^5^ Department of Material Science and Engineering College of Engineering Texas A&M University College Station Texas USA; ^6^ Center for Remote Health Technologies and Systems Texas A&M University College Station Texas USA

**Keywords:** hemostatic, nanomaterials, nanosilicates, protein corona

## Abstract

Tunable fabrication of inorganic two‐dimensional (2D) nanoclays is of substantial interest for fundamental studies and biomedical applications. Despite extensive work on inorganic biomaterials, layered silicate nanoclays remain a comparatively underexplored class of 2D systems. Here, we report an optimized hydrothermal method for the tunable fabrication of 2D layered magnesium nanosilicates (nSi). This approach yields disc‐like nanoclays with lateral dimensions of  ~20–50 nm and thickness of ~1–2 nm. The nanosilicates exhibit rapid cellular internalization, controlled therapeutic release in vitro, and the ability to form shear‐thinning, thixotropic gels. Proteomic analyses indicate the formation of a protein corona enriched in factors associated with blood coagulation, consistent with in vitro clotting assays showing a concentration‐dependent reduction in clotting time, with decreases of ~50% at higher doses. In an in vivo rat liver‐laceration model, nanosilicate treatment reduced clotting time by ~75% and blood loss by ~45%, compared with controls. Collectively, these findings establish a tunable route for fabricating 2D‐layered magnesium nanosilicates and suggest their potential utility in hemostasis and wound‐management applications.

## Graphical abstract

Tunable 2D magnesium nanosilicates form bioactive protein coronas, support cellular internalization, and promote hemostatic activity. These nanoclays enable shear‐thinning gel formation and reduce clotting time and blood loss, highlighting their potential for wound‐management applications.

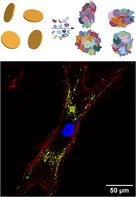

## 1 Introduction

Inorganic nanomaterials are increasingly recognized for their role in advancing regenerative medicine and theranostics [1, 2, 3]. Among them, dimensionality is a key design parameter, motivating significant interest in inorganic two‐dimensional (2D) nanomaterials [4, 5]. Compared with 0D, 1D, or bulk counterparts, 2D nanomaterials offer a high specific surface area and unique physicochemical properties, which have enabled the development of biomaterials such as transition metal dichalcogenides (TMDs), transition metal oxides (TMOs), metal carbides and nitrides (MXenes), layered double hydroxides (LDHs), and layered silicates (nanoclays). Analysis of the chemical structure of layered silicates suggests substantial untapped potential for accessing new compositions, shapes, and sizes of particles that have not yet been fully explored in biomedical engineering [6, 7, 8, 9, 10, 11, 12, 13, 14]. Such advances could, in principle, support the design of injectable therapies, printed electronics, bioprinted scaffolds, and delivery vehicles.

Nanoclays are mineralogically classified as phyllosilicates (“sheet silicates”), comprising over 100 naturally occurring silicate clay minerals. To date, only a subset has been explored in biomedical research, notably smectites (e.g., hectorite and montmorillonite) [15, 16, 17, 18] and kaolin group minerals (e.g., kaolinite and halloysite) [17, 19, 20, 21, 22]. Relative to other silicate or metallic nanoparticles, phyllosilicates exhibit attractive properties for drug delivery, adsorption, catalysis, and rheological modification, owing to their layered structure and defect‐rich crystallography [23, 24]. These features facilitate exfoliation into individual nanosheets with high aspect ratios, anisotropic morphology, and heterogeneous surface charge. Such characteristics have contributed to the development of kaolin‐impregnated combat gauze (QuikClot, Z–Medica Corporation) and smectite‐based rheological additives (Laponite, BYK Additives Ltd.; Bentone, Elementis Specialties). However, both synthetic and natural nanoclays commonly used in biomedical research offer limited tunability compared with other 2D inorganic nanomaterials, which constrains the design of layered silicate–based nanomedicines. This tunability can afford greater control over synthesizing nanomedicines with desired properties, especially for loading and releasing therapeutic agents, interacting with blood proteins, and using the nanomaterial directly as a therapeutic agent [25, 26].

A deeper understanding of synthesis–structure–property relationships is therefore needed to better exploit the potential of layered silicates. In this work, we use a hydrothermal approach to establish a tunable synthesis route for 2D magnesium nanosilicates. We characterize their structure, rheological behavior, drug‐delivery capabilities, hemostatic properties, and cytocompatibility. Finally, the hemostatic ability of the synthesized 2D nanosilicate was explored. This facile synthesis platform with further improvement in scale‐up process can be leveraged in future in a range of biomedical applications, including regenerative medicine, drug delivery, and additive manufacturing.

## 2 Results and Discussion

To design layered silicate nanomaterials, we developed a synthesis method to produce particles that mimic smectite clay minerals. These particles, referred to as magnesium nanosilicates (nSi), are distinct from smectite particles commonly reported in the literature, whose properties have not been optimized for biomedical applications. Because the unique characteristics of layered silicates arise directly from their structure and composition, precise control over synthesis is essential.

**FIGURE 1:**
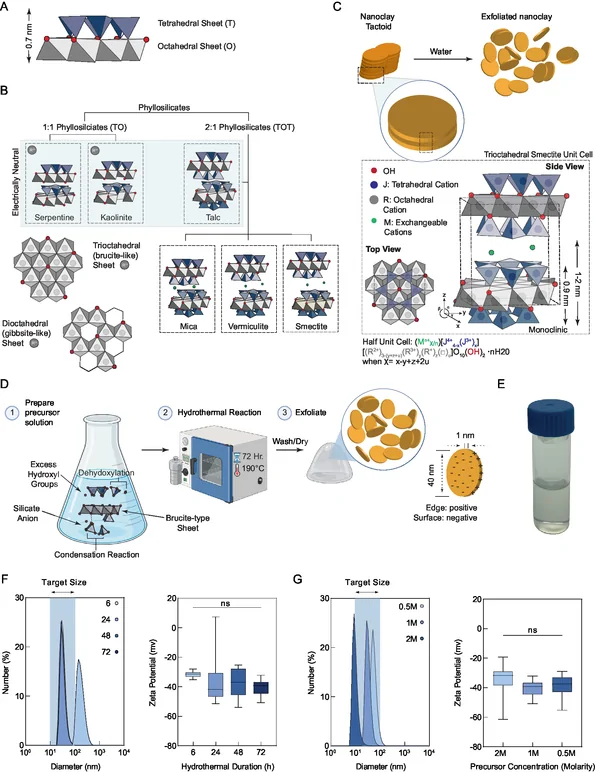
Structure, classification, and hydrothermal synthesis of layered nanosilicates. (A) Schematic of phyllosilicate layer architectures, illustrating 1:1 (TO), 2:1 (TOT), and 2:1:1 (TOT:O) arrangements formed by tetrahedral (T) and octahedral (O) sheets. (B) Classification of phyllosilicate clay minerals based on layer type, octahedral occupancy (dioctahedral vs. trioctahedral), and layer charge. (C) Illustration of tactoid formation and exfoliation of phyllosilicates into individual nanosheets, with the trioctahedral smectite unit cell shown. (D) Schematic of nanosilicate (nSi) synthesis, consisting of precursor formation, hydrothermal condensation (190 °C, 72 h), and exfoliation. (E) Representative colloidal suspension of magnesium nanosilicates (Mg‐nSi), prepared at 5% (w/v) in deionized water. (F) Hydrodynamic diameter and zeta potential of nanosilicates as a function of hydrothermal duration (6–72 h), measured by DLS and ELS, showing uniform size and stable negative surface charge for reaction times ≥24 h (mean ±  SD, *n* = 3; ns, not significant). (G) Effect of precursor concentration (0.5–2 M) on nanosilicates particle size and zeta potential, demonstrating size tunability with preserved surface charge (mean  ±  SD, *n* = 3; ns, not significant). Some of the icons used in the schematics are obtained from Biorender.com.

Phyllosilicates consist of planes of tetrahedrally and octahedrally coordinated cations, defined as tetrahedral (T) and octahedral (O) sheets, joined by covalent bonds (Figure 1A). The structural arrangement (number and type of sheets; i.e., 1:1/TO, 2:1/TOT, and 2:1:1/TOT:O) and composition (molecular ions; i.e., silicon tetrahedron: SiO_4_^−4^) heavily influence the properties of the clay mineral and are used to distinguish between mineral subgroups (Figure 1B). Smectites, a type of TOT clay mineral, have high cation‐exchange capacity, sorptive potential, and swelling behavior as a result of isomorphic substitution of lower valency cations or vacancies in tetrahedral and octahedral positions (denoted as J and R in Figure 1C) [25, 26]. The resultant net negative layer charge, typically within the range of 0.2–0.6 per unit formula, is weak enough to enable the exchange of hydrated cations, which compensate for the layer charge, across the interlayer space, which links adjacent layers (Figure 1C). These hydrated exchangeable cations give layered silicates their designation as “cationic clay minerals” and help maintain layer cohesion.

In general, smectites show high loading capacity for cationic therapeutics and can also adsorb anionic or nonpolar species at their edges, compensating for broken bonds at particle boundaries [23]. A key advantage of smectites is the relative ease of exfoliating tactoidal stacks into ultrathin (~1 nm) 2D nanoparticles or nanosheets (Figure 1C). Isolating individual nanosheets exposes the negative charge on the planar faces, promoting interparticle interactions and enabling shear‐thinning gel formation at low concentrations in deionized water [27]. Smectites are also attractive as synthesis platforms because they can form under moderate hydrothermal conditions with high phase purity. Notably, transition‐metal trioctahedral smectites, which do not occur naturally, have been synthesized hydrothermally; however, key particle parameters were either not reported or not suitable for biomedical use, motivating further optimization [28].

### 2.1 Hydrothermal Synthesis of Layered Silicates

To synthesize nanosilicates, we adapted a hydrothermal synthesis method, originally designed for the large‐scale manufacturing of magnesium silicates, a trioctahedral smectite [29]. Primarily, the synthesis parameters needed to be optimized from the original method to accommodate small batch synthesis in a nonindustrial setting (see Section 5). Fundamentally, the synthesis operates under the principle that nucleation of trioctahedral smectites is initiated by the condensation of silicate tetrahedra onto brucite‐like sheets [30]. As a result, the precipitation of nanosilicates is heavily reliant on the pH and availability of metallic and silicate ions.

The first step in this synthesis is the precipitation of silicate, in the form of SiO_3_^2−^, in an alkaline solution containing a water‐insoluble metallic compound. Both syntheses discussed here (see Section 5) incorporated some fraction of insoluble magnesium through a double‐replacement reaction with sodium carbonate. Sodium carbonate, additionally, has also been shown to aid in the formation of smectites as a mineralizing agent. Carbonate ions are used as a replacement for fluorine ions commonly used in other established syntheses to avoid the deleterious effects to the crystal structure as well as potential toxicity issues later [28, 31]. The presence of carbonate ions increases the availability of divalent metallic ions during synthesis while also participates in hydrolysis to establish pH conditions favorable for smectite formation [6, 32].

Brucite‐like sheets then form primarily through the olation reaction mechanism. Additional appropriately sized soluble metallic ions present during this step are expected to substitute into the crystal structure during formation by this same mechanism. To this end, a soluble lithium compound, lithium sulfate, is added to the solution to achieve isomorphous substitution and establish the characteristic layered charge.

The resulting suspension is transferred to a sealed Teflon vessel and heated at elevated temperature. Under hydrothermal conditions, the nuclei formed in the previous step grow, with carbonate ions continuing to participate in dissolution, hydrolysis, dehydroxylation, and condensation. Unreacted species are subsequently removed by dialysis, and nanosilicate crystals are obtained by drying and grinding the product into a fine powder. Individual nanosheets are prepared by exfoliation in deionized water (Figure 1D,E).

For biomedical applications, it is important that the synthesized particles preserve properties relevant to their use as rheological modifiers and delivery vehicles. Particle size and surface charge are known to be the key predictors of nanoparticle behavior in biological systems, influencing cellular internalization and trafficking, cytotoxicity, biodistribution, therapeutic loading, and rheological behavior [33]. We, therefore aimed for particles with diameters <100 nm to facilitate cellular uptake, while limiting cytotoxicity, and with a net negative surface charge from isomorphic substitution to support colloidal stability (ζ  >  ±30 mV) and promote endocytic uptake [23, 34, 35].

Hydrothermal duration or reaction time, strongly influences particle size, size distribution, morphology, and crystallization [36]. Therefore, we first optimize such factors by leveraging the parameters controlling nucleation and growth, specifically hydrothermal duration (6–72 h) and precursor concentration (0.5–2 M) (Figure 1F). Analysis of the hydrodynamic diameter and zeta potential of synthesized particles indicates that beyond 24 h, the hydrothermal duration has a negligible effect on both the zeta potential and particle size. Further, the hydrodynamic diameter and zeta potential of the synthesized nanosilicates agree well with previous measurements of commercially available Laponite nanosilicate. However, at 6 h, unreacted species appear to inhibit exfoliation, resulting in a larger particle size and opaque colloidal dispersion (Figure 1F). On this basis, we adopted a 72‐h duration to ensure sufficient time for particle growth and coarsening, while acknowledging that shorter times may also be feasible.

Precursor concentration or supersaturation, also has a substantial influence over nucleation mechanism, resulting in changes to particle size and distribution [36]. We, therefore varied the molarity of silicate and metal ion solutions from our original synthesis to be either halved (0.5 M) or doubled (2 M). Our analysis revealed that the zeta potential of synthesized nanoparticles did not significantly differ across these conditions. However, the hydrodynamic diameter appeared to decrease with increasing precursor molarity. The 2 M concentration resulted in ~20 nm decrease in particle size from approximately 30 to 10 nm, which only narrowly aligned with our target diameter range of 10–100 nm. Meanwhile, the larger particles, produced using the 0.5 M synthesis, adversely affected optical clarity and rheological properties. While potential morphological or crystal changes could have contributed to these differences, they were not investigated in this study (Figure 1G). Ultimately, we selected a synthesis duration of 72 h and a 1 M precursor concentration as the optimal conditions for producing magnesium nanosilicates (nSi).

### 2.2 Physical and Chemical Characteristics of Synthesized Nanosilicates

**FIGURE 2:**
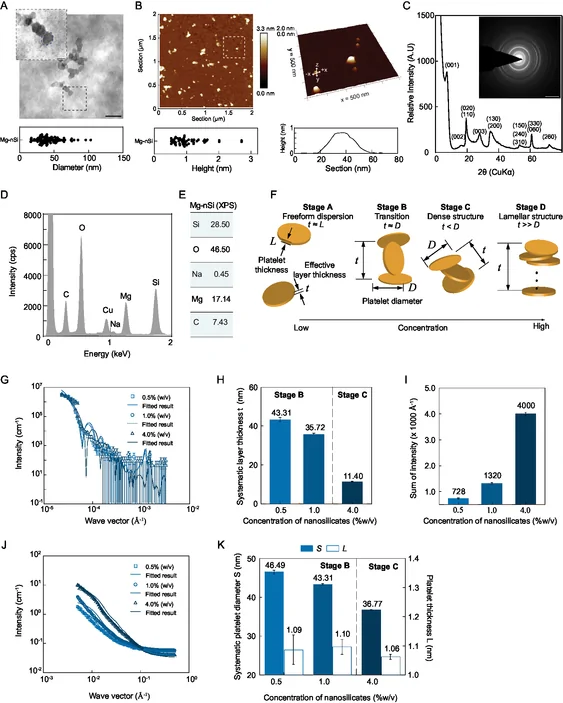
Morphology, structure, and concentration‐dependent assembly of nanosilicates. (A) TEM images of nanosilicates, showing disc‐like morphology (scale bar 100 nm); inset shows higher magnification (scale bar 25 nm). Particle diameter distribution indicates an average diameter of ~43 ±  17 nm. Scale bars: 100 and 25 nm. (B) AFM analysis of individual nanosheets. Height profiles indicate an average thickness of ~1.0 ± 0.5 nm, consistent with single‐layer nanosilicates. (C) XRD analysis, confirming a trioctahedral smectite structure; inset shows TEM‐SAED polycrystalline diffraction pattern. Indexed reflections correspond to expected lattice planes. (D) EDS spectrum, confirming elemental composition of nanosilicates, with dominant Si and Mg peaks. (E) XPS elemental analysis of nanosilicates, confirming surface composition consistent with layered magnesium silicates. (F) Schematic illustrating concentration‐dependent microstructural evolution of nanosilicates, transitioning from dispersed platelets to dense and lamellar assemblies. (G) USANS scattering intensity as a function of wave vector for nanosilicates at increasing concentrations, showing systematic structural evolution. (H) Quantified effective layer thickness extracted from USANS fits, demonstrating a concentration‐dependent decrease (mean  ±  SD, *n* = 3). (I) Integrated scattering intensity from 2D patterns as a function of nanosilicates concentration, reflecting increased structural ordering. (J) EQ‐SANS scattering profiles confirming nanosilicate assembly trends at higher *q*‐ranges. (K) Extracted platelet diameter (*S*) and thickness (*L*) as a function of concentration, showing reduced lateral dimensions with preserved nanosheet thickness (mean  ±  SD).

Following the synthesis, we evaluated the morphology, size, crystal structure, and elemental composition of the synthesized nanosilicates. Given the importance of nanoparticle size and morphology in both cellular interactions as well as intraparticle interactions, transmission electron microscopy (TEM) and atomic force microscopy (AFM) were used to verify these particle properties (Figure 2A,B). This revealed that platelet‐shaped 2D nanoparticles are approximately 20–50 nm in diameter and 1–2 nm in height. Generally, 2D morphology and high surface area are essential for facilitating therapeutic loading for delivery applications, mediating bio‐material interactions, and producing exotic electrical properties [12, 13]. Moreover, particle dimensions are highly indicative of edge‐site properties, which are responsible for the reactivity (functionalization, adsorption, and catalysis) and colloidal properties of smectites [37, 38].

X‐ray diffraction (XRD) was used to elucidate the crystal structure of smectites, with the diffraction pattern indicating that synthesized nanosilicates have a highly crystalline structure with peaks matching that of trioctahedral smectites (Figure 2C). In particular, the XRD pattern for glycolated nanosilicates exhibits a peak at 2*θ * =  6.059° corresponding to the basal reflection (001), the presence of which, alongside additional higher‐order basal reflections (002 and 003), indicates a well‐ordered crystal sample. This peak is of particular importance as it can be used as a surrogate for characterizing layer charge and swelling capacity. Using the (001) peak, the *d*‐spacing gives the distance between corresponding octahedral sheets of adjacent stacked nanoparticles, also known as the *c*‐axis lattice constant, which would include the interlayer space and crystal layer thickness (Figure 2C). To obtain better insight, ethylene glycol solvation was used to expand the interplanar space through the replacement of exchangeable cations with ethylene glycol molecules as a standard. This resolved to a length of approximately 14.5 Å or 1.45 nm, demonstrating swelling of the interplanar region to about 0.55 nm, aligning with known values for both smectites and vermiculites [39]. This level of swelling furthermore indicates a layer charge of approximately 0.6 per formula unit, and the formation of only weak hydrogen bonds to interlayer water molecules, which is characteristic of octahedral substitution [23, 37]. Additionally, the *d*‐spacing of the peak corresponding to the 060, 330 reciprocal *hkl* reflections at 1.5 nm (2*θ * =  ~60.93°) suggests synthesized particles are trioctahedral, as opposed to dioctohedral [23]. Dioctohedral smectites are identified by XRD through the vacancies in the crystal structure, given the evidence for layer charge and lack of evidence for vacancies, we can assume the successful substitution of lithium into the crystal structure. The nanosilicates sample does not exhibit any evidence of impurities, such as carbonates, which would have resulted in extraneous peaks and impeded the particles’ rheological characteristics [31]. Based on the XRD analysis, the observed swelling behavior corresponds to an estimated layer charge of ~0.6 per formula unit. ICP‐MS analysis showed consistent Li and Mg concentrations across nanosilicate samples, with Li ranging from 2.64 to 2.70 µg/g and Mg ranging from 108.3 to 112.4  µg/g, while Na levels remained comparatively low (1.12–2.21  µg/g). The comparatively higher Li/Mg ratio, together with the low Na content, supports Li‐for‐Mg substitution within the octahedral sheet as the primary source of layer charge formation. In contrast, Na is likely present mainly as an interlayer charge‐balancing cation contributing to electrostatic compensation and swelling behavior (Figure S1).

A comprehensive elemental analysis was carried out using energy‐dispersive spectroscopy (EDS) and X‐ray photoelectron spectroscopy (XPS) (Figure 2D,E). For nanosilicate particles, silicon and magnesium were found to be present at an atomic ratio of 4:2.4, resulting in an approximate atomic ratio of tetrahedral cations to octahedral cations ranging from 4:2.4 to 3.0, depending on lithium substitution (which cannot be measured by EDS) and magnesium‐ion intercalation. This is consistent with the ideal chemical formula of (M^*n*+^χ/*n*) [J^4+^_4−*x*_(J^3+^)_*x*_] [(R^2+^)_3−(*y* + *z* + *u*)_(R^3+^)_*y*_(R^+^)*_z_* (□)*_u_*] O_10_(OH)_2_·*n*H_2_0, where χ = x − *y* + *z* + 2*u*, J refers to the tetrahedral cations, and R refers to the octahedral cations (Figure 1C).

The microstructure evolution of synthesized nanosilicates upon the concentration change is hypothesized and illustrated in Figure 2F. At an extremely low concentration, the nanosilicate platelets are dispersed as isolated, individual entities. In this freeform‐dispersion stage, the effective layer thickness (*t*) of the nanosilicate microstructure is equivalent to the thickness (*L*) of a single platelet. As the concentration increases, electrostatic interactions between platelets occur, leading to the formation of loose “house‐of‐cards” microstructure. In this transitional stage, *t* approximates the diameter (*D*) of a single platelet. With the increase of nanosilicate concentration, platelets stack in a better‐organized manner due to the enhanced electrostatic interactions, which enables the formation of dense “house‐of‐cards” microstructure. Therefore, *t,* in this stage, is less than *D*. When the concentration is increased to an extremely high level, nanosilicate platelets in the system stack and align with each other, resulting in the generation of lamellar microstructure with a significantly high *t* value.

To validate the hypothesized microstructure evolution, ultra‐small‐angle neutron scattering (USANS) is leveraged first to explore the microstructures of nanosilicates at different concentrations. Herein, the stacked lamellae model [40, 41] (Equations (1) – (4) ) are applied to determine the *t* values with the key fitting parameters summarized in Table S1.

(1) $$I_{\text{stack}}\;\left(q\right)=2\pi \frac{P_{\text{stack}}\;(q)S_{\text{stack}}\;\left(q\right)}{q^{2}t}+\text{Background}$$

(2) $$P_{\text{stack}}\;\left(q\right)=\frac{2{\left(\rho_{p}-\rho_{s}\right)}^{2}}{q^{2}}\left(1-\cos\;q\;t\right)$$

(3) $$S_{\text{stack}}\;\left(q\right)=1+2\sum_{1}^{N-1}\left(1-\frac{n}{N}\right)\;\cos\;\left(qdn\right)\;\exp\left(-\frac{2q^{2}d^{2}\alpha \left(n\right)}{2}\right)$$

(4) $$\alpha \;\left(n\right)=\frac{C}{4\pi^{2}}\left(\ln\;\left(\pi n\right)+\gamma_{E}\right),\gamma_{E}=0.5772156649$$

where *q* is the scattering vector, Background is the scattering contribution from an incoherent background, *ρ*_p_ is the scattering length density of nanosilicate, *ρ*_s_ is the scattering length density of solvent (deuterium oxide), *N* is the number of stacked layers, *d* is the spacing between clusters, and *γ*_E_ is the Euler’s constant. Based on the scattering data in Figure 2G, the *t* values of nanosilicate platelets at three concentrations (i.e., 0.5%, 1%, and 4%) are calculated as shown in Figure 2H. At the lower concentrations (i.e., 0.5% and 1%), the *t* values are approximately 40 nm, which is close to the nanosilicate diameter, validating the formation of loose “house‐of‐cards” microstructure in the transition stage of Figure 2F.

When the concentration is increased to 4%, the *t* value decreases significantly to 11.40 nm, which is less than the nanosilicate diameter, showing the densification of “house‐of‐cards” microstructure and more tightly packed platelets in the system as illustrated in Figure 2F. Except for the effective layer thickness, the Caille parameter (*C*) also demonstrates the microstructure evolution, which describes the degree of smearing or broadening in the scattering profile due to thermal fluctuations or other forms of disorder within the lamellar stacks [42]. A *C* value approaching 0 indicates the formation of a well‐ordered or rigid lamellar system [43]. As shown in Figure S2, the *C* value at 4% is only 0.08, much lower than those at 0.5% and 1%. Therefore, a denser microstructure is formed at higher nanosilicate concentrations, which supports the microstructure evolution models in Figure 2F.

An extended *q*‐range small‐angle neutron scattering (EQ‐SANS) is then applied to further obtain the finer geometrical information of nanosilicates in the system at different concentrations. The 2D scattering patterns of the nanosilicates are summarized in Figure S3A. We performed the azimuthal analysis [44] to the patterns to evaluate their radial symmetry, as shown in Figure S3B. The uniform intensity distribution across the azimuthal angles proves the isotropic scattering in the patterns. By calculating the sum of intensity of all patterns (Figure 2I), 4% nanosilicate presents a significantly high total intensity (4000 Å^−1^), which demonstrates the formation of a larger and more organized microstructure at this concentration [45]. Then, the cylinder model [46] (Equations (5) and (6)) is applied to fit the measured intensity data (Figure 2J) in order to achieve two key geometries of nanosilicate platelet in the system: (1) effective platelet diameter (*S*) and (2) platelet thickness (*L*) as illustrated in Figure 2K.

(5) $$\begin{array}{cc} I_{\text{cylinder}}\;\left(q\right)= & \frac{\varphi_{\text{particle}}}{v_{P}}\;.\;\int_{0}^{\pi /2}{P_{\text{cylinder}}}^{2}\;\left(q,\alpha \right).\sin\;\alpha d\alpha \\ & \;+\text{Background} \end{array}$$

(6) $$P_{\text{cylinder}}\;\left(q,\alpha \right)=2\left(\rho_{p}-\rho_{s}\right)v_{p}\frac{\sin\left(\frac{1}{2}qL\;\cos\;\alpha \right)}{\frac{1}{2}qL\;\cos\;\alpha }\frac{J_{1}\left(\frac{1}{2}qS\;\sin\;\alpha \right)}{\frac{1}{2}qS\;\sin\;\alpha }$$

where *φ*_particle_ is the volume fraction of nanosilicate platelets, *v*_P_ is the volume of platelets, *J*_1_ is the first‐order spherical Bessel function, and *α* is the angle between the axis of nanosilicate platelet and the scattering vector. The parameters for fitting the EQ‐SANS data are summarized in Table S2. As shown in Figure 2K, nanosilicates at different concentrations have the constant platelet thickness of around 1 nm. However, two distinct regimes are observed when evaluating the effective platelet diameter. At lower concentrations (e.g., 0.5% and 1%), *S* approximates the diameter of synthesized nanosilicate platelets of ~40 nm, indicating the formation of a loose “house‐of‐cards” microstructure in Figure 2F. When the concentration increases to 4%, *S* decreases to 36.77 nm, which shows the microstructure densification.

In conclusion, the hydrothermal synthesis method of nanosilicates has proven effective in yielding smectite‐like nanoparticles. By optimizing synthesis parameters, such as precursor concentration and hydrothermal duration, nanosilicates with 2D discotic morphology, high specific surface area, and net negative charge were successfully achieved for magnesium nanosilicates. This method allows for generation of nanosilicate particles with similar chemical properties to commercially available nanosilicate Laponite [27] while maintaining the ability to modify the synthesis to achieve varying particle size, charge, and surface chemistry.

### 2.3 Nanosilicate‐Induced Thixotropy in Colloidal Gels

**FIGURE 3:**
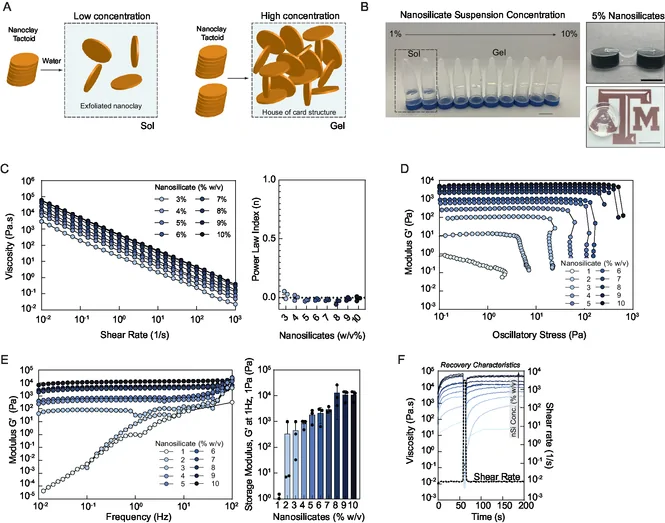
Concentration‐dependent rheological behavior of nanosilicate suspensions. (A) Schematic illustrating nanosilicate exfoliation and interparticle interactions, leading to a concentration‐dependent sol–gel transition. (B) Vial inversion test of nanosilicate suspensions (1%–10% w/v) demonstrating qualitative viscosity increase and sol–gel transition 24 h after preparation. Insets show self‐supporting behavior and optical clarity of a 5% (w/v) suspension (*n* = 3). (C) Shear‐rate sweeps showing shear‐thinning behavior across concentrations. Bar graphs summarize power‐law index (mean  ±  SD, *n* = 3). (D) Oscillatory stress sweeps used to determine storage modulus (*G*′) and yield stress as a function of concentration. (E) Frequency sweeps showing elastic‐dominated behavior at various concentrations. Bar graph reports *G*′ at 1 Hz (mean  ±  SD, *n* = 3). (F) Peak‐hold shear‐recovery tests simulating extrusion‐relevant shear, demonstrating rapid viscosity recovery following high‐shear deformation.

The thixotropic properties of nanosilicates (time‐dependent shear‐thinning and shear‐recovery), which make them very attractive for applications, such as injectable hydrogels [18] or 3D printing, are a result of inhomogeneous charge distribution, as discussed previously, and the resultant face–rim interactions [14]. Rheological characterization confirms the presence of directional interactions between particles, resulting in a sol–gel transition consistent with models of patchy colloidal systems, which should indicate long‐term colloidal stability [27]. At low concentrations, colloidal systems form arrested empty liquids (sols) which escalate toward a Wigner glass (gel) as concentration increases over time, the structure stabilizes into a house‐of‐cards glass (gel) through directional “T” interactions as opposed to long‐range interactions [27, 47, 48, 49] (Figure 3A,B).

Rheological analysis of nanosilicate suspensions, ranging from 1 to 10 percent weight by volume (% w/v), offers insights into determining the most suitable concentration for injectable applications. Nanosilicate suspensions were prepared in deionized water to facilitate dissolution of the tactoid structure and formation of the exfoliate or house‐of‐cards structure. Deionized water in particular was selected to allow for nanosilicate platelets to interact with each other, rather than with ions present in the solvent (such as for phosphate‐buffered saline or other physiological saline solutions). An important consideration for injectability is often shear‐thinning capacity as a measure of the viscosity under shear [50, 51]. An oscillatory shear‐rate sweep measures the presence and strength of reversible interparticle interactions, which enable a solid to flow under pressure. The shear‐rate sweeps were performed from 10^−3^ to 10^3^ s^−1^ using a parallel‐plate rheometer (Figure 3C). Shear‐rate sweeps of colloidal suspension nanosilicates demonstrate the presence of reversible cross‐linking and their shear‐responsive breakdown to impart injectability under shear. Specifically, concentrations as low as 3% (w/v) for nanosilicates demonstrate near‐perfect shear‐thinning behavior, the solutions below 3% did not show good viscosity.

Flow behavior is further characterized by the storage “elastic” modulus (*G*′) and yield stress. These viscoelastic properties are important to improve print fidelity, mechanical integrity, and cell shielding during extrusion [14]. The storage modulus is found from the linear viscoelastic region (LVER) of the curve obtained from an oscillatory stress sweep (Figure 3D) and frequency sweep (Figure 3E). The results indicate storage moduli ranging from 10 Pa to 10 kPa based on suspension concentration for magnesium nanosilicates. The yield stress represents the critical stress at which the colloidal gel begins transitioning from elastic to viscous behavior because of reversible bonds between particles. Yield strength values were obtained from the oscillatory stress sweep by identifying the oscillatory stress at the point where there is a distinct drop in the storage modulus. In all cases, the samples showed near‐Newtonian flow above the apparent yield stress. Again, yield values increase in accordance with nanosilicate concentration, ranging from 1 to 200 Pa for nanosilicates. This yield strength can be increased with the addition of a polymer component, but maintains a dose‐dependent increase in yield strength with increasing nanosilicate concentrations.

Additionally, a frequency sweep was used to further characterize colloidal interactions within the system and evaluate frequency‐dependent viscoelastic properties. Colloidal stability in water is crucial for various biomedical applications, ensuring the biocompatibility and efficacy of colloidal systems used in drug delivery, imaging, tissue engineering, diagnostics, and other biomedical technologies [16, 27, 52, 53, 54]. The loss modulus and complex viscosity are omitted for clarity, but the storage modulus indicates that at or above 3% (w/v), suspensions exhibit frequency‐independent elastic behavior, while suspensions below 3% (w/v) are frequency‐dependent (Figure 3E). Furthermore, in suspensions at or above 3% (w/v), the storage modulus dominates over the loss modulus for all frequencies assessed. The complex viscosity is dependent on the frequency, indicating a well‐structured system which is unlikely to aggregate or sediment. Suspensions below 3% (w/v) exhibit characteristics of a weakly structured system with shorter relaxation times, as the loss modulus dominates at low frequencies.

In order to characterize the shear‐recovery capacity of colloidal gels, suspensions underwent a peak hold test to assess recovery of gels after high shear to mimic extrusion. For applications in printing and in situ cross‐linking, near instantaneous recovery is required as this determines print fidelity and diffusion away from the injection site. The 5% and 6% (w/v) nanosilicates suspensions performed best with approximately 80% viscosity recovery after 180 s and 50% recovery with the first 10 s following the high shear block (Figure 3F). Suspensions below 5% (w/v) were able to recover at least 80% viscosity, but their recovery time was progressively slower as the concentration of nanosilicates dropped, thus indicating that with fewer particles, nanosilicates needed to migrate further to achieve an optimal arrangement capable of providing structure. Alternatively, higher concentrations (above 6% (w/v)) had recovery times comparable to 5% and 6% (w/v) suspensions but, likely due to steric hindrance, did not reach 80% viscosity recovery by the end of the test. However, their viscosity was still higher than that of less‐concentrated suspensions at any time point.

In summary, the unique properties of nanosilicates, including shear‐thinning behavior and self‐healing capabilities, make them highly promising for applications in injectable hydrogels and 3D printing. Rheological analyses, including shear rate sweeps, oscillatory stress sweeps, and frequency sweeps, reveal the viscoelastic properties and colloidal interactions within the system. Suspensions with higher viscosity and moduli are more likely to exhibit plug‐flow behavior during extrusion/injection, increasing cell shielding and print fidelity, while lower viscosity suspensions would be easier to inject [14, 55]. Therefore, nanosilicate concentration would need to be optimized based on the application and the other component materials in the engineered system. Moreover, peak‐hold tests demonstrate the self‐healing capacity of nanosilicate colloidal gels, with intermediate concentrations showing faster recovery rates. These findings highlight the potential of nanosilicates in biomedical and material science fields, offering versatile solutions for various applications requiring tailored rheological properties. Additionally, these properties recapitulate the rheological behavior of commercial Laponite nanoparticles and nanosilicate–polymer composite systems [56], which highlights the ability to tune nanosilicate synthesis, while maintaining injectability and material mechanics.

### 2.4 Evaluation of In Vitro Hemo‐ and Cytocompatibility of Nanosilicates

**FIGURE 4:**
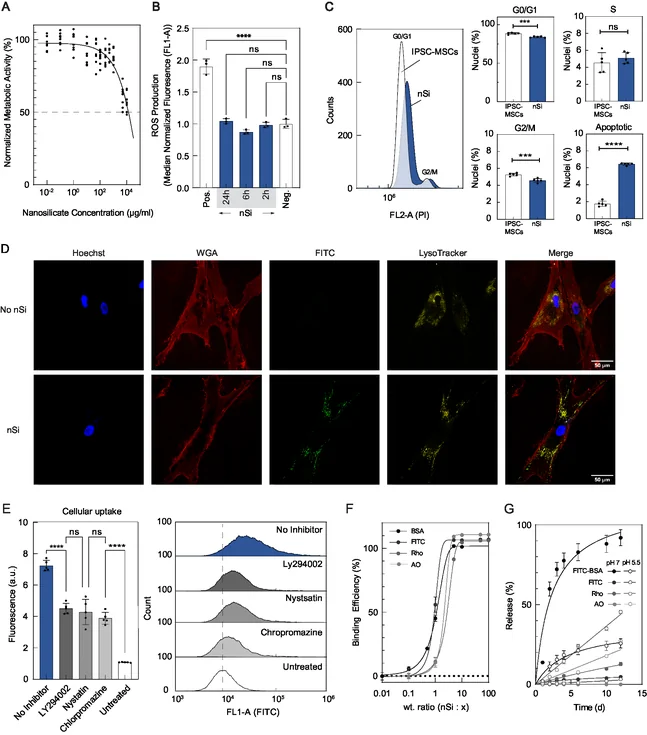
Cytocompatibility, cellular interactions, and cargo handling of nanosilicates. (A) Cell viability of iPSC‐MSCs following nanosilicate (nSi) exposure (0–10 mg mL^−1^, 24 h), quantified by alamarBlue assay and normalized to untreated controls. Nonlinear regression was used to determine half‐maximal inhibitory concentration (IC_50_). (B) Reactive oxygen species (ROS) production following nanosilicates treatment (2, 6, and 24 h), quantified by fluorescence imaging. No significant increase relative to untreated controls; H_2_O_2_ (100 µM) served as positive control (mean ±  SD, *n* = 3; *p < 0.05; **p < 0.01; ***p < 0.001; ****p < 0.0001). (C) Cell‐cycle analysis by flow cytometry (PI staining) after nanosilicates treatment (50 µg  mL^−1^). Distributions across G0/G1, S, G2/M, and apoptotic cells populations are shown (mean  ±  SD, *n* = 3; *p < 0.05; **p < 0.01; ***p < 0.001; ****p < 0.0001). (D) Confocal microscopy showing intracellular uptake of FITC‐labeled nanosilicates (50  µg  mL^−1^, 24 h) and colocalization with lysosomes (Lysotracker). Nuclei and membranes stained with Hoechst and wheat‐germ agglutinin (WGA), respectively. (E) Endocytosis inhibition study of FITC‐BSA‐loaded nanosilicates using flow cytometry. Uptake is reduced by inhibitors of clathrin‐mediated endocytosis (chlorpromazine), micropinocytosis (LY294002), and caveolar (nystatin) pathways, indicating multipathway internalization (mean  ±  SD, *n* = 3; *p < 0.05; **p < 0.01; ***p < 0.001; ****p < 0.0001). Right: schematic of cell–particle interactions. (F) Binding efficiency of model cargos—acridine orange (AO), rhodamine B (Rho), fluorescein isothiocyanate (FITC), and FITC‐tagged bovine serum albumin (FITC‐BSA) to nanosilicates as a function of mass ratio, showing near‐complete binding at ≥5:1 (nanosilicates:cargo) (mean  ±  SD, *n* = 3). (G) Cargo‐release profiles of small molecules and FITC‐BSA at pH 7.4 and pH 5.5, demonstrating sustained and pH‐responsive release over 12  days.

Cytocompatibility testing plays a crucial role in the development and translation of nanoparticle‐based technologies [34]. The metabolic activity of cells was monitored with increasing concentration of nanosilicates, using alamarBlue assay (Figure 4A). Active cells reduce resazurin, the dye in alamarBlue, causing a change in absorbance. A decrease indicates reduced metabolic activity, correlating with diminished cellular viability. Cells treated with varying concentrations of exfoliated nanosilicates (0–10 mg/mL) for 24 h were assessed to identify the half‐maximal inhibitory concentration (IC_50_). The assay revealed an IC_50_ value of ~10 mg/mL for nanosilicates, which is more than 10 times higher than other 2D inorganic nanomaterials [4]. This value is similar to that previously determined for commercial Laponite at ~4 mg/mL.

In order to better evaluate the biochemical effects of nanosilicates on cellular function, we analyzed reactive oxygen species (ROS) production (Figure 4B). Intracellular ROS is assessed using flow cytometry and the general oxidative stress indicator, CM‐H2DCFDA, which undergoes oxidation upon exposure to ROS, resulting in fluorescence emission proportional to the intracellular ROS levels. Utilizing the concentration of 50 µg/mL (~IC_50_), cells were treated with nanosilicates for 2, 6, and 24 h. In all cases, we see no significant difference between ROS production in treated wells versus our untreated cells, while the positive control (cells treated with 100 μM H_2_O_2_) showed a significant increase in ROS production.

To further evaluate the effects on broader cellular function, we analyzed cell growth and division. Cell‐cycle analysis was conducted using propidium iodide (PI) dye, where untreated cells served as the negative control, and the treatment group was exposed to 50 μg/mL nanosilicates for 3 days. Cell‐cycle analysis was performed using flow cytometry. The peaks in the histogram represent distinct populations of cells with varying DNA content, corresponding to different phases of the cell cycle (Figure 4C). We observed that nanosilicate treatment resulted in an increase in apoptotic cells by approximately 5%, aligning with the cell‐viability results. In live cells, nanosilicates do not appear to induce cell‐cycle arrest, as evidenced by a similar percentage of each cell‐cycle phase in treated cells compared to controls. Additionally, this reveals a slight reduction in the G0/G1 phase and an increase in the S phase, suggesting enhanced cellular proliferation in response to treatment.

We further assessed cellular internalization of nanosilicates via confocal microscopy (Figure 4D). Cells treated with fluorescently tagged nanosilicates were imaged via confocal microscopy to visualize nanoparticle internalization; lysosome staining via lysotracker overlaps strongly with tagged nanoparticles, suggesting endocytosis by cells and strong co‐localization with lysosomes. To investigate the internalization mechanisms, an endocytosis inhibition assay was conducted using flow cytometry. Specifically, the effects of endocytosis inhibitors, including chlorpromazine (clatherin‐mediated), LY294002 (micropinocytosis), and nystatin (caveolar‐mediated), were assessed on cellular uptake of fluorescently tagged nanosilicates (Figure 4E). The positive control reflects the fluorescent intensity of internalized nanosilicates in the absence of any inhibitor, while cells without nanosilicates serve as the negative control. Significant differences observed between our nanosilicates groups and controls suggest inhibition of nanoparticle uptake in response to inhibition of each tested endocytosis mechanism.

The high specific area of 2D nanoparticles greatly enhances their ability to act as delivery vehicles. To evaluate the loading of differently charged drugs, we compare the adsorption of small‐molecule fluorescent dyes: AO—acridine orange (cationic dye), FITC—fluorescein isothiocyanate (anionic dye), and Rho B—rhodamine B (amphoteric dye). Utilizing a fixed concentration of dye (10 μg/mL) and various concentrations of exfoliated nanosilicates (0–1000 μg/mL), the binding efficiency of the nanosilicates was indirectly examined through the fluorescence measurements of the unloaded dyes. In all cases, regardless of charge, nearly ~100% binding efficiency is attained at approximately a nanosilicate/dye mass ratio of 5:1 or 20 wt% nSi, where the fluorescence of the supernatant after centrifugation matched that of the negative control, which contained no dye. Similarly, the loading of differently sized therapeutics, as modeled by the adsorption of a small molecule, FITC, and a model large molecule, bovine serum albumin (BSA), yielded the same approximate mass ratio of 5:1. Indicating that the mass of nanosilicates must be at least five times that of the therapeutic of interest for 100% binding efficiency (Figure 4F), which is consistent with previous results for synthetic nanosilicates [57]. Interestingly, the dye’s electrostatic interactions with the particles led to the neutralization of particle charge at intermediate concentrations (100–500 µg/mL), resulting in a visually precipitation out of solution. This phenomenon suggests that fully loaded particles tend to lose their colloidal stability.

Nanosilicates can be protonated and deprotonated to alter electrostatic interactions and enable pH‐responsive therapeutic release and degradation [13, 58]. This aspect can be utilized to take advantage of acidic environments such as the lysosome or tumor microenvironment, to increase therapeutic release. To evaluate this property for synthesized nanosilicates, we quantify the therapeutic release of molecules with different charges and sizes in physiological pH (pH = 7) and acidic pH (pH = 5.5) (Figure 4G). After fully loading the nanosilicates using the ratio determined previously, cumulative release was calculated based on the quantity of dye present in the supernatant after the specified amount of time. In the case of both acridine orange (cationic dye) and rhodamine B (amphoteric dye), the result showed enhanced release of their cargo. The loaded FITC (anionic dye) appears unaffected by the change in pH of its environment. Collectively, this suggests that protonation selectively affects the release of positively charged particles from nanosilicates likely through neutralization of the negative surface charge. Additionally, BSA, which has a net negative charge and 66.5 kDa molecular mass, experiences greater release in neutral pH as opposed to acidic pH. Moreover, BSA is the only cargo, which demonstrates initial burst release followed by sustained delivery, which is likely correlated with cargo size. In summary, nanosilicates appear the most suited for loading with cationic cargo in order to take advantage of the pH‐responsive nature of nanosilicates. Nevertheless, nanosilicates have the capacity to provide sustained delivery of a wide variety of therapeutic cargo regardless of both charge and size.

In addition to cytocompatibility, we sought to characterize the hemocompatibility of nanosilicates. Lysis of erythrocytes, which can be measured through the concentration of free heme and hemoglobin released from ruptured cells, can cause dangerous changes in blood viscosity and contribute to thrombosis [59]. Regardless of the route of administration, nanoparticles often come into direct contact with blood, especially when used in medical applications like drug delivery, imaging, implants, or hemostatic materials, and so, establishing a safety profile is essential. Hemolysis was assessed via the addition of exfoliated nanosilicates (1 µg/mL–1 mg/mL) suspended in a deionized water and PBS mixture following exposure to a fixed concentration of washed erythrocytes isolated from human blood. After 1 h incubation, centrifugation was used to separate the solid fraction from the free proteins. 0.1% Triton X and 1× PBS were used as positive and negative controls, respectively, to calculate the relative percentage of hemolysis, in accordance with existing literature [60]. As per the ASTM E2524‐22 standard, our findings indicate that nanosilicates demonstrated nonhemolytic properties with all concentrations up to and including 1 mg/mL (Figure S4).

### 2.5 Understanding Protein Corona Formation on Nanosilicates

**FIGURE 5:**
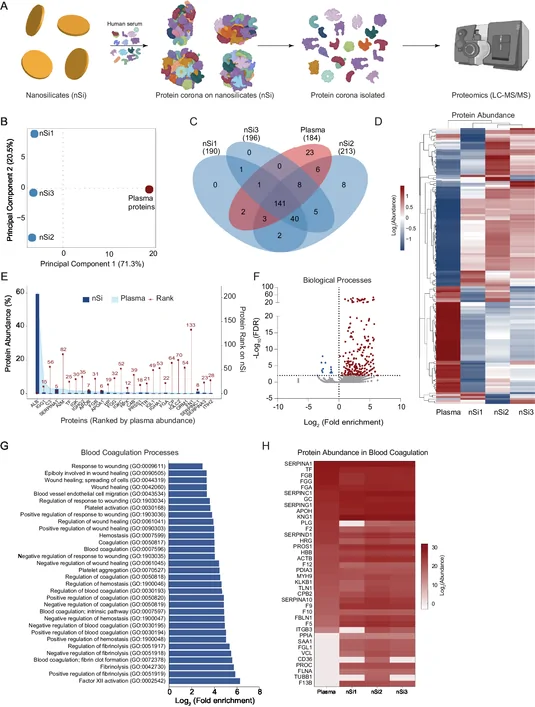
Protein corona formation on nanosilicates in human plasma. (A) Workflow for protein corona analysis, in which nanosilicates (nSi) are incubated with human serum, followed by isolation of the tightly bound protein corona and LC–MS/MS proteomic analysis. (B) Principal component analysis (PCA) of protein corona composition showing distinct clustering of nSi1, nSi2, and nSi3 relative to plasma proteins. PC1 and PC2 explain 71.3% and 20.5% of the variance, respectively (91.8% total). (C) Venn diagram illustrating overlap and uniqueness of tightly bound proteins: nSi1 (190), nSi2 (213), nSi3 (196), plasma (184), with 141 proteins shared across all conditions. (D) Hierarchical clustering of *Z*‐score normalized protein abundance, revealing nanosilicate‐enriched and competitively displaced plasma protein clusters. (E) Protein rank‐abundance analysis showing preferential adsorption and enrichment of specific proteins on nanosilicates surfaces relative to plasma. (F) Gene ontology (GO) biological process‐enrichment analysis, highlighting strong enrichment for blood coagulation (GO:0007596; 17‐fold enrichment, Log_2_FE = 4.06, −Log_10_FDR = 34.55). (G) Log_2_ (fold enrichment) analysis of coagulation‐related pathways, showing enrichment of Factor XII activation, fibrinolysis, and fibrin clot formation. (H) Heatmap of coagulation‐associated protein abundance, demonstrating selective enrichment of key blood coagulation proteins on nanosilicates surfaces. Some of the icons used in the schematics are borrowed from Biorender.com.

When introduced to blood circulation, the nanosilicates readily bind plasma proteins on its surface, forming a layer of protein corona. To define the governing protein corona formation on nanosilicates, we performed quantitative, label‐free proteomic profiling of tightly bound plasma proteins isolated following plasma exposure (Figure 5A, Supplementary Datasets 1–4). Principal component analysis (PCA) demonstrated reproducibility of the nanosilicate protein corona (Figure 5B). PC1 captured 71.3% of the total variance, while PC2 accounted for 20.5%, together explaining 91.8% of the dataset variability. The tight clustering of independent nanosilicate replicates, coupled with their clear separation from plasma controls, demonstrates that protein adsorption is driven by nanosilicate surface chemistry and physicochemical features. This supports the formation of a compositionally defined and consistent protein corona.

To further study the corona composition, Venn diagram analysis revealed distinct yet overlapping protein populations across nanosilicates formulations (Figure 5C). Specifically, 190 proteins were identified on nSi1, 213 on nSi2, and 196 on nSi3, compared to 184 proteins in plasma controls. Importantly, a conserved core of 141 proteins was shared across all nanosilicates samples, indicating stable, high‐affinity protein association that is largely independent of batch‐to‐batch variability. This conserved corona suggests the existence of energetically favorable binding motifs on the nanosilicate surface that preferentially recruit specific plasma proteins. Hierarchical clustering of normalized protein abundance profiles further resolved the corona into two functionally distinct clusters (Figure 5D). The first cluster consisted of nanosilicate‐enriched proteins (positive *Z*‐scores, red), while the second comprised competitively displaced proteins (negative *Z*‐scores, blue), dominated by high‐abundance plasma constituents. The persistence of a stable, enriched cluster highlights that nanosilicates selectively retain proteins with strong interfacial binding, despite competitive plasma conditions.

Ranked‐abundance analysis provided additional insight into corona composition (Figure 5E). While albumin remained the most abundant protein overall, high‐molecular‐weight kininogen (KNG1) exhibited a notable enrichment, advancing from rank 39 in plasma to rank 2 in the nanosilicate corona. Given its role in the contact activation system, its enrichment across the nanosilicate surface suggests that the protein corona includes factors relevant to hemostasis‐related processes. However, the present analysis is limited to protein identification and relative abundance and does not evaluate activation of downstream coagulation cascades.

To evaluate the functional consequences of this corona architecture, we performed gene ontology (GO) enrichment analysis of the nanosilicates‐bound proteome (Figure 5F). Biological processes associated with blood coagulation (GO:0007596) exhibited 17‐fold enrichment (Log_2_FE = 4.06, −Log_10_FDR = 34.55), with comparable enrichment observed for coagulation and hemostasis pathways. Importantly, regulatory processes, including regulation of blood coagulation and regulation of hemostasis, were also strongly enriched, indicating that the protein corona supports both activation and control of coagulation‐related processes. Detailed pathway‐level analysis (Figure 5G) revealed pronounced enrichment of Factor XII activation, fibrinolysis, and fibrin clot formation, alongside both positive and negative regulatory nodes within the coagulation network. Enrichment of platelet activation, response to wound, and wound healing‐associated processes further suggest that the nanosilicates protein corona orchestrates a coordinated hemostatic response that integrates clot formation with tissue‐repair mechanisms. This balanced enrichment profile indicates that nanosilicates have the potential to promote efficient yet regulated hemostasis, a critical requirement for safe clinical translation. Finally, heatmap analysis of coagulation‐related proteins (Figure 5H) confirmed selective enrichment of key regulators, including KNG1, SERPINA1, FGB, FGA, and FGG, on nanosilicates relative to plasma.

In conclusion, nanosilicates reproducibly assemble a functionally specialized, coagulation‐enriched protein corona through selective and competitive protein interactions. These results suggest that the nanosilicates are able to engage in controlled contact‐system activation, as well as potential for supporting wound healing and tissue regeneration. These findings position nanosilicates as a rationally engineered bio‐interface with strong potential for high‐impact translational applications in hemostasis and regenerative medicine.

### 2.6 In Vitro and In Vivo Hemostatic Ability of Nanosilicates

**FIGURE 6:**
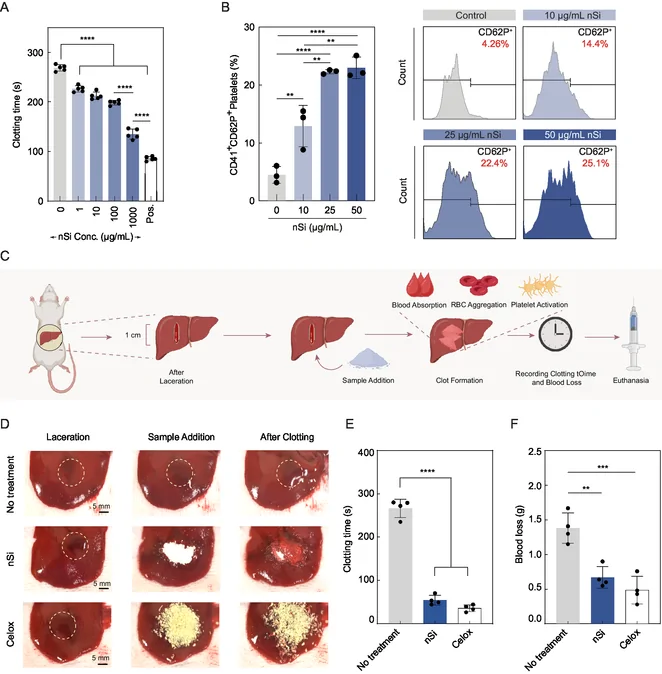
In vitro and in vivo hemostatic performance of nanosilicates. (A) Whole‐blood clotting assay with human blood, assessed by vial inversion. Data are represented as mean  ±  SD (*n* = 5,*p < 0.05, **p < 0.01; ***p < 0.001; ****p < 0.0001). Kaolin at 1 mg/mL is a positive control. (B) Effect of nanosilicates on platelet activation. Quantification of CD41^+^CD62P^+^ platelets following exposure to nanosilicates. Representative flow‐cytometry histograms show CD62P surface expression on unstimulated platelets and platelets treated with 10, 25, or 50 µg/mL nanosilicates. Data are presented as mean ± SD (*n* = 3, *p < 0.05; **p < 0.01; ***p < 0.001; ****p < 0.0001; ns, not significant (*p* > 0.05)). (C) Schematic of the rat liver‐laceration model used to evaluate in vivo hemostatic efficacy. (D) Representative images of liver laceration following injury, material application, and clot formation for no treatment, nanosilicates, and Celox. (E) Clotting time measured in the liver‐laceration model. Nanosilicates significantly reduce clotting time relative to no treatment and the value is comparable to Celox (mean  ±  SD, *n* = 4; *p < 0.05; **p < 0.01; ***p < 0.001; ****p < 0.0001). (F) Blood loss quantified in the liver‐laceration model, showing significant reduction with nanosilicate treatment compared to no treatment (mean ± SD, *n* = 4; *p < 0.05; **p< 0.01; ***p  < 0.001;****p < 0.0001). Some of the icons used in this figure are obtained from Biorender.com.

Based on our proteomic analyses revealing enrichment of coagulation‐associated proteins, we next evaluated the hemostatic activity of nanosilicates using a whole‐blood clotting assay [61]. We observed a statistically significant, concentration‐dependent reduction in clotting time, indicative of procoagulant activity (Figure 6A). The result indicated that nanosilicates, even at a low concentration of 1 µg/mL, was able to reduce clotting time by ~15%, compared to no treatment. Though Kaolin performed better in reducing clotting time at same concentration as nanosilicates (1 mg/mL), application of nanosilicates demonstrated approximately ~50% reduction in the in vitro clotting time, which could be crucial to manage blood loss for better clinical patient outcome, especially in the prehospital care management. While uncontrolled systemic exposure could pose a thrombogenic risk, this dose‐dependent procoagulant property suggests that the nanosilicates can be strategically exploited for localized hemostatic applications, consistent with prior reports on nanomaterial‐based hemostats and embolic agents [19, 62, 63, 64, 65].

To further delineate the cellular mechanisms underlying nanosilicate‐driven coagulation, we investigated their ability to activate platelets (Figures 6B, S5, and S6). Platelets are central regulators of primary hemostasis, and upon activation undergo morphological remodeling, surface adhesion, and degranulation, thereby amplifying coagulation signaling. A well‐established marker of platelet activation is the surface upregulation of P‐selectin (CD62P). Freshly isolated platelets were incubated with nanosilicates, and CD62P expression was quantified by flow cytometry. Nanosilicate treatment induced a robust, concentration‐dependent increase in platelet activation. Exposure to 10 µg/mL nanosilicates increased the fraction of CD62P^+^ platelets to 14.4%, compared with 4.26% in unstimulated controls. Further increases in nanosilicate concentration resulted in progressive enhancement of CD62P expression, reaching 22.4% at 25 µg/mL and 25.1% at 50 µg/mL. Collectively, these data demonstrate that nanosilicates actively promote platelet activation in a dose‐dependent manner, complementing their protein corona–mediated enrichment of coagulation factors. This result is aligned with that seen previously in our work with commercial‐grade nanosilicates [66].

To directly assess functional hemostatic performance in vivo, we evaluated nanosilicate powder using a rat liver‐laceration model, which is a well‐established animal model of hemorrhage and enables macroscale visualization of hemostasis under physiologic conditions (Figure 6C–F). Following standardized liver incision, nanosilicate powder was applied directly to the wound site, and clotting time and blood loss were recorded. The treatment application was standardized by mass (i.e., 50 mg of nanosilicates or Celox applied to the wound site). Celox has a lower density than nanosilicates, and so a larger volume of sample was applied for these groups while maintaining a consistent mass of sample. Macroscopic imaging demonstrated that nanosilicates powder rapidly localized to the bleeding site, forming a stable physical barrier that resisted dissolution and wash‐off by blood flow (Figure 6D). In untreated animals, clotting time was approximately 4.5 min. In contrast, application of nanosilicates powder reduced bleeding cessation time to ~70 s, corresponding to a ~75% reduction in clotting time, comparable to the clinically used hemostat Celox (Figure 6E). Celox is a widely used commercial hemostatic agent delivered in a powder form. This particular hemostatic material was utilized in order to maintain consistency of the material form factor and application technique, rather than introducing additional effects from impregnated gauzes or scaffold structures. Consistent with these results, untreated animals exhibited a blood loss of 1.38 ±  0.22 g, whereas nanosilicates treatment reduced blood loss to 0.76  ±  0.13 g, representing a ~45% reduction (Figure 6F). This reduction in clotting time and blood loss are comparable, albeit slightly lower, than previously observed in a nanosilicate‐foam composite (~80% and ~56%, respectively) [66], highlighting the value of incorporating nanosilicates into composite materials to achieve strong hemostatic ability.

Collectively, these results demonstrate that nanosilicates exhibit dose‐dependent hemostatic activity when locally applied at therapeutic levels. Further, the nanosilicates can be administered either in a suspension (observed in the in vitro clotting time) or as a dry powder (observed in the in vivo study), highlighting the modular use and application of this material. In addition to injectability, ability to form a physical barrier at the site of injury, the synthesized nanosilicate demonstrated hemostatic properties. Though an in‐depth biocompatibility, biodistribution study, and improved hemostatic efficacy are indispensable for translation application, this strategy to fabricate 2D nanosilicates with hemostatic properties provides a platform to build on future studies in prehospital trauma care and wound management.

## 3 Limitations

The studies presented in this work have several limitations. Although the hemostatic action of the nanosilicates represents a significant improvement over the untreated group, the blood loss and clotting time measurements are higher than that of Celox, a commercial hemostatic agent. As discussed previously, the sample addition in the in vivo study was standardized based on mass, rather than volume. Celox and nanosilicate powders have different densities, and so, this could be a probable reason for observing reduced clotting time and blood loss between nSi powder and Celox (Figure 6E,F). A multifactorial study exploring varying mass, volumes, and concentrations of nanosilicates in a wound cavity should be explored in the future to fully understand the hemostatic potential of this material.

Perhaps most importantly, these studies focus on the short‐term evaluation of the nanosilicate platelets and have not extended into long‐term study. Regarding material stability, we have evaluated the chemical and rheological properties of nanosilicates and freshly prepared nanosilicate colloids. These materials demonstrate strong mechanical stability, evidenced by shear‐independent moduli. However, long‐term characterization of the nanosilicates was not performed. Although we see strong stability of the colloids, it is possible that the nanosilicates may begin to precipitate out of solution or lose their mechanical integrity, especially in the presence of adsorbed proteins or interaction with ions present in blood and other body fluids.

Although we have established cytocompatibility, blood–material interactions, and in vivo hemostatic ability of the nanosilicate platelets, we have not yet probed the long‐term consequences of this material. Our in vivo study was limited to a short‐term nonsurvival study; however, further investigation into the systemic biocompatibility, biodegradation, and subsequent effects following successful hemostasis should be investigated to understand the potential material changes or toxicity under prolonged physiological conditions. Additionally, in vitro testing, including our analysis of the protein corona formation, was limited to immediate and short‐term effects, which fails to provide an understanding of how these conditions may evolve over time. We have yet to explore long‐term effects of the nanosilicates on cytocompatibility or how prolonged exposure may impact cellular growth and reproduction. Further, we have not yet explored the stability of the protein corona over time, which may have an impact on the hemolytic activity of the nanosilicates as well as the physiological stability of the colloid. Although outside the scope of this study, these characterizations are essential to establish a robust view of the nanosilicates’ potential for clinical translation.

Due to the hemostatic nature of the nanosilicates, there is a risk of the particles entering blood vessels and forming a thrombus after hemostasis. The small size of nanoparticles allows them to traverse biological barriers and potentially disrupt cellular environments [59]. Although a different route of exposure, inhaled silica nanoparticles have been known to enter the bloodstream and accumulate in the reticuloendothelial system [67], suggesting that the nanoparticles may be able to travel through the bloodstream. However, incidental nanoparticles are unlikely to cause thrombus formation as a concentration of >1 µg/mL is needed to trigger clotting compared to blood alone (Figure 6A). Additionally, shear stresses present in blood vessels are typically less than 5 Pa [68, 69], which is within the LVER and lower than the yield stress of the nanosilicates when at concentrations of at least 3% (Figure 3D), suggesting that nanosilicate/blood colloids would not flow or fragment at this stress. In the in vivo animal model, nanosilicates were applied as dry powder, where a stable plug was observed. Hence, it is unlikely that the nanosilicates would fully dissolve into the blood prior to forming a clot, making the embolization of the nanosilicates into the bloodstream unlikely. As owing to high local concentrations, forming clot before dispersing does not provide a direct evidence of minimizing risk, related to systemic distribution, thrombogenicity, and systemic accumulation, validating the in vivo biocompatibility and assessing for any systemic distribution of the nanosilicates is essential to understanding the safety profile of this nanomaterial A thorough investigation of the systemic biodistribution, accumulation, and clearance of the nanosilicates should be conducted prior to clinical translation.

## 4 Conclusion

In this work, we establish a facile, tunable, synthetic strategy for the fabrication of magnesium nanosilicates. We report the controlled synthesis of magnesium nanosilicates, a layered silicate architecture that structurally mimics hectorite, with magnesium and lithium occupying octahedral lattice sites. Through precise control of hydrothermal synthesis parameters, we achieved 2D nanosilicates with well‐defined disc‐like morphology, nanoscale dimensions (20–50 nm in lateral size and 1–2 nm in thickness), high phase purity, and a swelling capacity of ~0.45 nm. Nanosilicates exhibited shear‐independent mechanical stability, shear‐thinning behavior, and thixotropic rheological properties that are critical for injectable, printable, and conformal biomedical formulations. Beyond materials synthesis, we demonstrate that nanosilicates exhibit robust short‐term biological compatibility, including high cellular viability and hemocompatibility within defined concentration windows. Importantly, nanosilicates assemble a functionally specialized protein corona enriched in coagulation‐related proteins, suggesting a mechanism for interaction with hemostatic pathways. Overall, the hemostatic property demonstrated by the synthesized nanosilicates supports the tunability of the developed platform, which can be used to design future therapeutic intervention strategies.

## 5 Materials and Methods

### 5.1 Materials and Reagents

Nanoparticle synthesis involved the acquisition of magnesium sulfate heptahydrate (Prod. No. M1880) and sodium silicate solution (Prod. No. 1.05621) from Sigma–Aldrich (St. Louis, MO, USA). Zinc oxide (Cat. No. 422741000) was obtained from Thermo Fisher Scientific (Waltham, MA, USA). Lithium sulfate monohydrate (Cat. No. 131265) was procured from BeanTown Chemical (Stoughton, MA, USA). Sodium carbonate (Cat. No. BDH9284) was purchased from BDH Chemicals (Dorset, UK), and sodium hydroxide (Prod. No. S24000) was sourced from RPI (Mount Prospect, IL, USA). Magnesium silicate (Laponite) was obtained from BYK Additives Ltd (Wesel, Germany). Cell‐culture experiments utilized human‐induced pluripotent stem cell–derived mesenchymal stem cells (iPSC‐MSCs) provided by Dr. Carl Gregory’s Lab at the Institute for Regenerative Medicine, Texas A&M Health [70]. Cells were cultured in α‐minimal essential media (alpha‐MEM) (HyClone, GE Sciences) supplemented with 10.0% FBS (Atlanta Biologicals) and 1% penicillin/streptomycin (100 U/100 μg/mL; Gibco). Hematology testing utilized citrate–phosphate–dextrose–adenine (CPDA‐1) bovine blood acquired from TAMU CVM ‐ Veterinary Medical Park. In vitro assays incorporated ROS indicator CM‐H2DCFDA (Cat. No. C6827) and LY294002 (Cat. No. PHZ1144) from Thermo Fisher Scientific, PI (Prod. No. P3566) and RNase (Prod. No. 12091‐021) from Invitrogen, nystatin (Prod. No. N6261) and chlorpromazine (Prod. No. 31679) from Sigma–Aldrich, and alamarBlue (Cat. No. DAL1025) from Invitrogen. Loading studies utilized rhodamine B (Prod. No. A13572), FITC‐dextran (Prod. No. FD2000S), and albumin‐fluorescein isocyanate conjugate (Prod. No. A9771) from Sigma–Aldrich. Acridine orange (Prod. No. A1301) was acquired from Invitrogen (Carlsbad, CA, USA). Confocal images were obtained using Lysotracker Red DND‐99 (Cat. No. L7528) and wheat‐germ agglutinin 633 conjugate (Cat. No. 29024‐1) from Invitrogen, and Hoechst 33342 (Cat. No. 40046) from Biotium (Fremont, CA, USA).

### 5.2 Synthesis of Nanosilicates

The synthesis procedure presented was adapted for the substitution of metal cations at autogenous pressure from an industrial‐scale hydrothermal synthesis method originally for magnesium silicates under moderate pressure [29]. The molar ratio of starting materials containing selected tetrahedral (J) cations to octahedral cations (R) were kept at 4:3 to align with the ideal chemical formula of (M^*n*+^χ/*n*) [J^4+^_4−*x*_(J^3+^)_x_] [(R^2+^)_3−(*y*+*z*+*u*)_(R^3+^)*_y_* (R^+^)*_z_* (◻)*_u_*] O_10_(OH)_2_ ·*n*H_2_0, when χ = *x* − *y* + *z* +2*u*. The cations considered were magnesium and zinc (as divalent R^2+^ cations) and lithium (as monovalent R^+^ cations). ◻ denotes vacancy in crystal lattice. For each formulation, metal salts (magnesium sulfate, zinc oxide, and lithium sulfate) were dissolved into distilled water at 0.875 M for R^2+^ and 0.125 M for R^+^. 1 M sodium carbonate is added dropwise at a rate of 60 mL/h to form brucite‐like sheets at an atomic ratio of 1:1 (Na to R). Afterward, (1.5 M) sodium silicate solution is added at an atomic ratio of 4:3 (J to R) dropwise at a rate of 80 mL/h. The solution was stirred by a magnetic stirrer overnight or until homogenous. Next, 75 mL of prepared solution was poured into stainless‐steel autoclaves with PTFE‐lined vessels with a capacity of 100 mL. The autoclaves containing the solutions were placed in the oven under static conditions at 190 °C for 72 h for all syntheses unless otherwise noted. The contents from the autoclave were then washed using dialysis for 2–3 days and dried at 110 °C overnight in a vacuum oven to isolate nanosilicate crystals. Colloids are prepared via suspension of powdered nanosilicate, prepared via mortar and pestle, in DI water; colloids were vortexed and then bath sonicated and left undisturbed overnight. If inhomogeneity remained, colloids were then probe sonicated at 20% power (10 s on/10 s off) for 5 min on ice and once again left undisturbed overnight.

### 5.3 Nanoparticle Characterization

XRD (Bruker D8 Endeavor) was used to determine crystalline structure of powdered nanosilicates. XRD was performed with a copper radiation source emitting X‐rays at a wavelength of 1.540598 Å. Data collection was performed in *θ*–2*θ* mode with a scan range of 2°–80° 2*θ* and a data collection speed of 30 min. The experiment was conducted at room temperature with a slit width of 1 mm, and samples were mounted flat for analysis. Powder samples of nanosilicates were prepared by ethylene glycol vapor solvation at 60 °C overnight. The *d*‐spacing, or measure of the distance between adjacent lattice planes, was calculated using Bragg’s law, given by Equation (7):

(7) nλ=2dsin(ϑ)

where *n* is the order of the diffraction peak, *λ* is the incident wavelength, *d* is the *d*‐spacing, and *θ* is the angle of incidence. Diffraction patterns were then verified using selected area diffraction conducted with the FEI TECNAI G2 F20 ST FE‐TEM.

Elemental composition of nanosilicate samples was determined using inductively coupled plasma–mass spectrometry (ICP–MS, PerkinElmer NexION 300D ICP‐MS equipped with a 4DXCi autosampler (Elemental Scientific Inc., USA)) at the ICP–MS Core Facility, Texas A&M University (Figure S6). The samples were acid‐digested to dissolve the silicate matrix. Briefly, 0.5 mL of nanosilicate suspension were transferred into metal‐free polypropylene digestion tubes, followed by the addition of 3 mL concentrated trace‐metal grade nitric acid (HNO_3_, 65%) and 1 mL concentrated hydrofluoric acid (HF). Samples were heated at 60 °C for 1 h and subsequently cooled to room temperature. The digested solutions were diluted to a final volume of approximately 20 mL using ultrapure deionized water, and the total mass of each solution was recorded. A subsequent 200‐fold dilution was prepared by transferring 50 μL of the digest into 9.95 mL of 2% (v/v) HNO_3_. The resulting solutions were analyzed directly without further dilution. Operating conditions for the ICP–MS included an RF power of 1600 W, plasma argon flow rate of 18.0 L/min, auxiliary argon flow rate of 1.20 L/min, and nebulizer argon flow rate of 0.96 L/min. Samples were introduced at a flow rate of 1 mL/min using a concentric nebulizer coupled to a cyclonic spray chamber. Data acquisition was performed in peak hopping mode with five points per peak, a dwell time of 50 ms, and 20 sweeps per reading. The instrument utilized Ni/Ni/Al sampler, skimmer, and hyper‐skimmer cones and was operated using Syngistix Software (Version 3.4). The measured elemental molar ratios (Li/Mg and Na/Mg) were used to quantify octahedral substitution and layer charge using Equation (8). Assuming a trioctahedral smectite framework with six octahedral sites per formula unit, lithium substitution was calculated as

(8) $$f_{\mathrm{Li}}=\;\frac{Li/\mathrm{Mg}}{1+Li/\mathrm{Mg}}$$

(9) $$Li_{\mathrm{pfu}}=6*\;f_{\mathrm{Li}}$$

(10) $$Mg_{\mathrm{pfu}}=6-\;Li_{\mathrm{pfu}}$$

Because Li^+^ substitutes for Mg^2+^ in the octahedral sheet, each substitution contributes one unit of negative layer charge. Therefore, the ICP–MS‐derived layer charge was estimated as

(11) $$\text{Layer charge }\left(\mathrm{pfu}\right)=Li_{\mathrm{pfu}}$$

The ζ potential and hydrodynamic size of nanosilicates solutions were initially measured with the Zetasizer Nano ZS (Malvern Instrument) furnished with a He–Ne laser at 25 °C. Nanosilicates suspensions were exfoliated in DI water overnight and diluted to achieve a mean count rate between 100 and 500 kcps (approximately 100 µg/mL). Dilutions were probe sonicated at 20% power for 5 min (10 s on/10 s off) on ice directly before reading. Reported means were calculated from averaging the number peaks from three separate reaction batches (five tests each).

AFM (Bruker Dimension Icon Nanoscope) and TEM (JEOL 1200 EX TEM and FEI TECNAI G2 F20 ST FE‐TEM) was performed to verify the size and shape of the individual nanosilicate sheets. For both AFM and TEM, a dilute solution of exfoliated nanosilicates were prepared in deionized water at 10 and 500 µg/mL, respectively. The silicon wafer (AFM) and carbon grids (TEM) were prepared via drop casting and allowed to dry. For AFM, nanosilicate thickness was acquired from the topograph obtained via peak force tapping, and the data were analyzed using Nanoscope Analysis software. For TEM, an accelerating voltage of 100 kV using the JEOL 1200 EX TEM and 200 kV using the FEI TECNAI G2 F20 ST FE‐TEM was used to determine the morphology of nanosilicates. The FEI TEM was also utilized to acquire chemical compositions via point analysis conducted with an EDS detector. XPS (Omicron DAR400) was, additionally, utilized to acquire atomic percentages present in nanosilicate powders.

### 5.4 Small‐Angle Neutron Scattering (SANS)

USANS experiments were performed at the Spallation Neutron Source (SNS) at the Oak Ridge National Laboratory (ORNL) to characterize the overall microstructures of synthesized nanosilicates at 0.5%, 1%, and 4% (w/v). The nanosilicate samples were prepared per the aforementioned protocol by replacing DI water with deuterium oxide (D_2_O, 99.9 atom % D, Sigma–Aldrich, Burlington, MA) to improve neutron‐scattering contrast. The sample‐to‐detector distances were set at 2.13 m. A double‐bounce, perfect‐crystal diffractometer setup was used and neutron wavelength of first harmonic at *λ* = 3.6 Å to provide a comprehensive *q*‐range from 3 ×  10^−5^ to 1  ×  10^−3^   Å^−1^. The nanosilicate samples were loaded into 1 mm path‐length quartz cells, sealed to maintain deuteration, and measured at ambient temperature to minimize background noise. Additionally, the EQ‐SANS diffractometer [71] at the SNS was used to further probe the nanosilicate microstructures. Two specific sample‐to‐detector distances (2.5 and 4.0 m) were implemented along with the minimum wavelength settings of *λ*_min_ =  2.5 and 10.0 Å, respectively, in order to achieve an extended *q* *‐*range from 5 × 10^−3^ to 6 × 10^−1^ Å^−1^. The choppers were operated at a frequency of 60 Hz to modulate the neutron beam. The *w* placed in 2.0 mm quartz cells and measured under ambient conditions. Data processing involved the correction of raw scattering profiles, instrumental background, and sample cell contributions using the standard reduction software developed at the ORNL. The intensity data were normalized to absolute units (cm^−1^) using a porous silica standard [72] to ensure the consistency across measurements. The SasView (https://www.sasview.org/), an open‐source software package, was applied to further analyze the data to characterize and interpret the microstructure evolution at different nanosilicate concentrations.

### 5.5 Rheological Characterization

Rheological characterization, performed using a TA Instruments Discovery Hybrid Rheometer (DHR‐2) equipped with a 20‐mm parallel plate geometry, measured the flow and deformation behavior of nanosilicate colloids at 25 °C. Nanosilicate colloids (1%–10% weight by volume (w/v) in deionized water) were prepared as described previously. Approximately, 150 µL of each was loaded onto the bottom plate, excess sample being removed at the trim gap, before lowering fully to the geometry gap of 400 µm. Oscillatory shear‐rate sweeps were performed between 10^−3^ and 10^3 ^s^−1^ to determine the power law index, *n*, via fitting of the power law model. Given the linearity of the logarithmic experimental data starting just after 0.001 and continuing to 1000 s^−1^, the power law model was used to quantify the shear‐thinning behavior of the colloidal gels within these shear values (see Equation (12)).

(12) $$\eta =K\gamma^{n‒1}$$

where *η* is the viscosity, *K* is the flow consistency index, *γ* is the shear rate, and *n* is the power law index. The power law index indicates the shear‐thinning behavior, with values closer to “0” acting as a perfect shear‐thinning fluid and values closer to “1” exhibiting more Newtonian behavior. Oscillatory stress sweeps were conducted from 0.1 to 1000 Pa at 1 Hz to confirm yield stress and storage modulus. Frequency sweeps were performed at 1 Pa (in the LVER) from 10^−2^ to 10^2^ Hz for viscoelastic characterization of the colloidal solutions. Finally, peak‐hold tests were performed starting at 10^−2^ s^−1^, dropping to 3000 s^−1^, then returning to 10^−2^ s^−1^ at time intervals of 60, 5, and 180 s, respectively.

### 5.6 Drug Loading and Release Kinetics

Drug loading and release studies were used to determine loading efficiency and release profiles. To evaluate these properties for differently charged drugs, we compared the loading and release of small molecule fluorescent dyes: AO, FITC, and RhoB. In the loading study, various concentrations of exfoliated nanosilicates (0–10 mg/mL) were suspended in PBS with 100 μg/mL dye. After 1 h incubation at 37 °C, suspensions were centrifuged at 18 000*g* for 10 m. The fluorescence of a 100‐µL aliquot was measured to indirectly determine binding efficiency (AO: 500/540, FITC: 495/519, RhoB: 546/568 [excitation/emission wavelength (nm)]). Release was then conducted by loading the particles at 100%. After the particles were loaded using the technique described previously, unloaded dye was measured, and particles were resuspended in either PBS (pH: 7) or PBS modified by hydrochloric acid (pH:5.5). At the specified timepoints, the suspensions were centrifuged, the supernatant fluorescence measured, and replaced with fresh PBS or acidic PBS solution.

Loading and release for differently sized molecules by comparing FITC to BSA. Protein loading on nanosilicates was determined using a µBCA assay. Again, various concentrations of exfoliated nanosilicates (0–1 mg/mL) were suspended in PBS with 100 μg/mL BSA and incubated for 1 h at 37 °C. Following incubation, the samples were centrifuged to separate the nanosilicates from the supernatant containing unattached protein. The concentration of unattached BSA in the supernatant was then quantified using the µBCA assay according to the manufacturer’s instructions. Release was quantified by tagging BSA with FITC and measuring the fluorescence of released BSA as was done previously for the small molecule dyes.

### 5.7 In Vitro Cell Culture Assays

In vitro assays were completed with cell populations (iPSC‐MSCs) under P5; cell passaging was completed as required (when flasks reached ~70% confluency) with 0.5% trypsin–EDTA. Media was exchanged every 2–3 days. Cells treated with nanosilicates were exposed to a freshly prepared solution of exfoliated nanosilicates (10 mg/mL in DI water), which was then diluted to 50 µg/mL in completed alpha‐MEM media. Exfoliated nanosilicates were prepared by UV sterilization at 40 mJ/cm^2^ and suspended in either nuclease‐free water or syringe‐filtered DI water. Fluorescently tagged nanosilicates were prepared by co‐incubating with FITC‐BSA for 1 h and washed via centrifugation in PBS thrice. Assays utilizing flow cytometry were run using the BD Accuri C6 flow cytometer at 35 µL/min until 10 000 events were captured in triplicate within the gated cell population.

Metabolic activity and cellular viability were measured via the AlamarBlue (Thermo Scientific) assay utilizing a Cytation 5 Imaging Reader, per the manufacture's protocols. IPSC‐MSCs were treated with exfoliated nanosilicates (0–10 mg/mL) for 24 h at 37 °C. The water content in each treatment case was controlled by preparing 10X solutions of exfoliated nanosilicates for dilution in alpha‐MEM for each concentration.

For evaluation of ROS production, iPSC‐MSCs were cultured in a 6‐well plate to ∼70% confluency and then treated with 50 µg/mL exfoliated nanosilicates for 2, 6, and 24 h at 37 °C, respectively. Then, cells were washed with PBS and incubated with 1 μM ROS indicator (CM‐H2DCFDA) diluted in serum‐free media. At 15 min, positive controls were treated with 100 μM H_2_O_2_ directly into the wells. After 30 min, cells were washed with PBS twice, trypsinized, spun down at 800*g*, and then resuspended in PBS for flow cytometer analysis.

To assess the impact of nanosilicates on the cell cycle, iPSC‐MSCs were first serum‐starved for 24 h to synchronize the cell populations and then treated with exfoliated nanosilicates (50 μg/mL) for 72 h. Following treatment, the cells were trypsinized and fixed in ice‐cold 70% ethanol. Fixed cells were centrifuged to form pellets, washed with PBS to remove residual ethanol, and resuspended in 40 μg/mL PI and 100 μg/mL RNase. The cells were incubated in this solution at 37 °C for 30 min to allow for sufficient DNA staining. Poststaining, the cells were kept at 4 °C until analysis. Flow cytometry was performed to analyze the cell‐cycle distribution, with data acquisition and analysis conducted using FCS Express software by De Novo Software.

For endocytosis‐inhibition analysis, cells were serum‐starved for 4–12 h before treatment, then washed with PBS and treated with inhibitors for clathrin‐mediated endocytosis, caveolar‐mediated endocytosis, and micropinocytosis (35 μM chlorpromazine hydrochloride, 10 μM nystatin, or 100 μM LY294002, respectively) (Sigma–Aldrich) at 37 °C for 30 min. After this pretreatment, cells were treated with fluorescently tagged nanosilicates (50 μg/mL) and incubated for a further 60 min. Subsequently, the cells were washed with PBS twice, trypsinized, and then resuspended in PBS. Particle uptake was then analyzed via flow cytometry. The negative control is untreated iPSC‐MSCs, while the positive control is iPSC‐MSCs treated with nanosilicates but no inhibitor.

Additionally, the uptake of nanosilicates was visualized utilizing a Leica SP8 confocal microscope. iPSC‐MSCs were seeded into a 2‐well chambered μ‐slide and cultured until reaching ~50% confluency. Again, cells are starved 4–12 h before treatment with fluorescently tagged nanosilicates. After 24 h, cells were incubated with 50 nM LysoTracker Red DND‐99 in alpha‐MEM for 30 min at 37 °C to stain lysosomes. Cells were then washed with PBS and fixed using 4% PFA in PBS for 10 min. For membrane and nucleus staining, fixed cells were incubated for 10 min at room temperature with 1 µg/mL far red Wheat Germ Agglutinin (CF633 WGA from Biotin) and 1 µg/mL Hoechst dyes, respectively, washing with PBS after staining. Confocal images were then collected the same day to maximize fluorescence.

### 5.8 Hemolysis Assay

A hemolysis assay was used to evaluate interactions between nanosilicates and red blood cells. Citrated human blood was obtained ethically from donors with informed consent under Texas A&M University Institute Review Board (IRB): 2022‐0501D. Washed erythrocytes were isolated from human blood by centrifuging repeatedly at 1700*g* for 15 min and diluted in PBS to create a 5% suspension. Exfoliated nSi (1 µg/mL–1 mg/mL) in deionized water was combined with 500 µL PBS, and the final volume adjusted to 1 mL with deionized water. Each concentration of nanosilicate was thus suspended in a mixture of 500 µL PBS and 500 µL deionized water. The positive control was 0.1% Triton X. The negative control was PBS. In 1.5 mL Eppendorf tube, samples or controls are mixed with erythrocyte suspension in a 1:1 volume ratio (100 µL each sample, 200 μl total) and incubated for 60 min at 37 °C. After centrifugation of the tubes at 1000 rpm for 5 min, 100 µL of supernatant was taken for the reading. The Cytation 5 Imaging Reader (BioTek Agilent Technologies, USA) is used to measure the absorbance of the supernatant at 540 nm. The representative images were also taken. The hemolysis was calculated using the following Equation (13).

(13) $$\mathrm{Hemolysis}\;\left(\%\right)=\left(\frac{A_{\text{sample}}-\;A_{\mathrm{neg}}}{A_{\mathrm{pos}}-\;A_{\mathrm{neg}}}\right)*100$$

### 5.9 Protein Corona

Protein corona on nanosilicates was investigated by incubating nanosilicates in 1 mL of human plasma at a concentration of 1 mg/mL for 1 h at 37 °C. Following incubation, the nanosilicates–protein corona complex was centrifuged at 10 000 × *g* for 10 min to remove unbound proteins. The resulting pellet was washed once with 1× PBS and centrifuged again at 10 000 × *g* for 10 min to isolate the hard protein corona. The tightly bound proteins were eluted from the nanosilicates by incubation in 1% SDS for 30 min, followed by centrifugation at 7 000×*g* for 10 min. The supernatant containing the eluted proteins was subjected to methanol–chloroform precipitation to recover the proteins. Briefly, four volumes of methanol and one volume of chloroform were added to the SDS eluate, followed by three volumes of deionized water. The mixture was vortexed after each addition and centrifuged at 14 000 × *g* for 5 min to achieve phase separation. The upper aqueous phase was carefully removed, leaving the protein‐containing interphase undisturbed. Four volumes of methanol were then added, and the samples were centrifuged at 14 000 × *g* for 10 min to pellet the proteins. Precipitated proteins were air‐dried and resuspended in sterile PBS. Protein concentration was quantified using the Pierce Dilution‐Free Rapid Gold BCA Protein Assay Kit (Thermo Fisher Scientific), in which 10 µL of each sample was mixed with 200 µL of the working reagent, incubated for 5 min, and absorbance was measured at 480 nm using a Tecan Infinite 200 Pro M Plex plate reader.

For proteomic analysis, 5 µg of purified protein from each sample was analyzed by liquid chromatography–tandem mass spectrometry (LC–MS/MS) using an Ultimate 3000 nano‐LC system coupled to an Orbitrap Fusion Tribrid mass spectrometer (Thermo Scientific). 1 µL of each sample was injected and separated on a 150 × 0.075 mm nanoLC column (Waters nanoEase M/Z Peptide BEH C18, 130 Å, 1.7 µm particle size) at a flow rate of 0.300 µL/min. The total run time was 60 min, with the following gradient: 2% B (98% acetonitrile, 2% water, 0.1% formic acid) for equilibration; ramp to 45% B at 37 min; ramp to 90% B at 40 min and hold until 46 min; ramp down to 2% B at 47 min and hold until 60 min. Eluent was introduced by nano‐electrospray ionization (nano‐ESI) at 2450 V. Mass spectra were acquired in positive ion mode at a resolution of 120 000 (m/z 200) over an *m*/*z* range of 400−1600. MS/MS spectra were acquired by higher‐energy collisional dissociation (HCD) at a fixed collision energy of 28%, using a precursor isolation window of 1.6 *m*/*z*, with fragment ions detected in the ion trap.

Raw data were processed using Proteome Discoverer v2.4 (Thermo Scientific). Protein identification and relative abundance data were exported as tab‐delimited files and analyzed in R (v4.5.1). Data were curated using the readr, dplyr, tidyr, and tibble packages across experimental conditions (nSi1, nSi2, nSi3, and plasma). PCA was performed on scaled protein abundance data using the prcomp() function, with visualization using ggplot2 and nonoverlapping labels implemented via ggrepel. Protein overlap across conditions was assessed using Venn diagram and grid packages. Hierarchical clustering was performed on *Z*‐score‐normalized protein abundance data using Euclidean distance and complete linkage, and heatmaps were generated using pheatmap. Protein rank–abundance relationships were evaluated by ranking proteins according to relative abundance and visualized using ggplot2. GO biological process‐enrichment analysis was performed on proteins identified in the nanoparticle‐associated corona using curated GO annotations. Statistical significance was assessed using a hypergeometric test, with multiple‐testing correction applied using the Benjamini–Hochberg false discovery rate (FDR) method. GO enrichment results were visualized as volcano plots, and enrichment of blood coagulation–related processes and corresponding protein heatmaps were generated using GraphPad Prism 8.4.

### 5.10 In Vitro Coagulation Assays

Coagulation assay was used to evaluate interactions between nanosilicates and the human blood. Citrated human blood was obtained ethically from donors with informed consent under Texas A&M University Institute Review Board (IRB): 2022‐0501D. The clotting time was measured using an inversion test. Exfoliated nanosilicate samples (0–1 mg/mL) and Kaolin (1 mg/mL) were prepared in deionized (DI) water. To prepare the test samples, these solutions (10X) were mixed with recalcified human blood (at a ratio of 9 parts whole blood to 1 part 0.1 M CaCl_2_), achieving a final concentration of 1X in a 1‐mL tube. The tubes were then incubated at 37 °C and inverted to a 90° angle every 15 s to check for clot formation. Clotting time was recorded when no visible blood flow was observed. 1 mg/mL Kaolin solution served as a positive control.

### 5.11 Platelet Activation Assay

The platelet‐activating potential of nanosilicates was assessed by flow cytometry. Human platelets were isolated according to Abcam’s Isolation of Human Platelets protocol. Briefly, 100 µL of the platelet suspension was aliquoted into microcentrifuge tubes and incubated with increasing concentrations of nanosilicates (10, 25, and 50 µg/mL) for 1 h at room temperature. Following incubation, samples were centrifuged at 750 ×*g* for 10 min and washed twice with PBS. Platelets were subsequently fixed in 2% paraformaldehyde for 30 min at room temperature, washed twice with PBS, and resuspended in 100 µL of FACS buffer (PBS supplemented with 1% BSA). Fixed platelets were stained with CoraLite Plus 647 antihuman CD41 (Proteintech; CL647‐65173) and antihuman CD62P‐FITC (Proteintech; FITC‐65587) antibodies for 30 min at room temperature in the dark. After staining, platelets were washed with FACS buffer, resuspended in 500 µL of the same buffer, and analyzed using a MACSQuant Analyzer 16. Unstimulated platelets served as the negative control.

### 5.12 In Vivo Experiments

All experiments performed in this study were conducted in accordance with the animal use protocol (IACUC2022‐0010D and IACUC2024‐0301D) approved by the Texas A&M University Institutional Animal Care and Use Committee. A total of 12 Sprague Dawley (SD) rats (3–6 months old, 200–500 g) were randomly distributed without any experimental bias into three experimental groups, including no treatment, nanosilicate (nSi), and Celox, where four rats with equal sex ratio (2M, 2F) were used for each experimental group. In vivo experiments with *N *= 4 were performed as 4 is the minimum sample size with the fewest animals necessary to run any statistical test, where the biological variation between different sex with equal ratio was taken into consideration. Post‐anesthesia with isoflurane and oxygen, the rats were placed in a supine position on the heating blanket to maintain physiological body temperature at 37 °C. Then, artificial tear gel was applied to avoid the dryness of the eyes, and after shaving the abdominal hair, the surrounding area was disinfected with 70% ethanol and 4% chlorhexidine gluconate (MP Biomedicals, Catalog # 0219136025). No treatment group serves as a negative control, and Celox acts as a clinical positive control.

The liver‐laceration study was performed using a modified established protocol [66, 73]. After exposing the liver by making an incision in the abdomen, the serous and body fluids were wiped out using a surgical nonwoven gauze (Medline Industries, USA). Then, a preweighed gauze was carefully placed below the liver, and an incision of ~2 cm length was performed to induce bleeding. 50 mg of sterilized samples was then immediately applied to the wound site, and clotting time and blood loss were quantified after the bleeding was completely arrested. The blood loss was measured using Equation (14). During the surgical procedure, representative images were captured, and after the rats were euthanized by injecting sodium pentobarbital (150 mg/kg), afterward.

(14) $$Blood\;\mathrm{loss}\;=\;m_{1}\text{‒}\;m_{0}$$

where *m*_1_ is the weight of nonabsorbent surgical gauze after clotting, and *m*_0_ is the initial weight of nonabsorbent surgical gauze.

### 5.13 Statistical Analysis

Statistical Analysis was determined using GraphPad 9.0. Statistical analysis was performed using either the one‐way ANOVA with post‐hoc Tukey HSD (when comparing three or more experimental groups) or a two‐tailed *t*‐test (when comparing two experimental groups); in both cases, *p < 0.05 is considered significant. Significance between samples is represented by *p < 0.05, **p < 0.01, ***p < 0.001, ****p < 0.0001.

## Author Contributions

A.L.K., K.A.S., and A.K.G. conceived the original idea of the project. A.L.K. lead study design, performed experiments, analyzed data, and prepared the manuscript. A.L.K., S.E.M., S.B., and K.A.S. assisted in blood compatibility study and in vitro cellular studies. V.R. assisted in animal experiments. W.H. and Y.J. performed small‐angle neutron scattering (SANS) measurements in collaboration with C.D. and W.R.C. from the Oak Ridge National Laboratory. K.K. and S.C. performed proteomics studies and analyzed the processed data in consultation with Dr. Klaudia I. Kocurek from the Mass Spectrometry Facility, Department of Chemistry at the Texas A&M University and Dr. Bryan Tomlin from the ICP‐MS facility, Department of Chemistry at Texas A&M University. S.R. and S.F. performed flow cytometry experiments. S.B. and V.R. performed in vitro and in vivo blood‐clotting experiments. A.K.G. provided funds, instruments, and supervised the project. All the authors commented on the results and participated in the writing and critical revision of the manuscript.

## Funding

This study was supported by the U.S. Department of Defense (W81XWH2210932).

## Conflicts of Interest

Provisional patent (TAMUS 6539, Provisional patent) is submitted on this technology.

## Supporting information

Supplementary Material

## Data Availability

All data needed to evaluate the conclusions in the paper are present in the paper and/or the Supporting Information. All raw data files are deposited in Zenodo data repository (https://doi.org/10.5281/zenodo.21865658).
