# Supplementary material for: Tunable Fabrication of 2D‐Layered Magnesium Nanosilicates With Hemostatic Properties
Source: Small Sci. 2026 Sep 9;6(9):e70374. doi: 10.1002/smsc.70374 (PMC13574045; doi:10.1002/smsc.70374)
Supplement: Supplementary file 1 [file SMSC-6-e70374-s001.pdf]

Supporting Information

Tunable Fabrication of 2D Layered Magnesium Nanosilicates with Hemostatic Properties

Anna L. Keller[1], Kanwar Abhay Singh[1], Saptarshi Biswas[1], Kavita Kadu[1], Sarah E. Miller[1], Weijian Hua[2], Sridevi Conjeevaram[1], Vaaridhi Ramanuja[1], Shounak Roy[1], Samantha Foster[3], Changwoo Do[4], Wei-Ren Chen[4], Yifei Jin[2], and Akhilesh K. Gaharwar[1,3,5,6,]*

[1]Department of Biomedical Engineering, College of Engineering, Texas A&M University, College Station, TX 77843, USA

[2]Mechanical Engineering Department, University of Nevada Reno, Reno, Nevada 89557, USA

[3]Interdisciplinary Program in Genetics, Texas A&M University, College Station, TX 77843, USA

[4]Neutron Scattering Division, Oak Ridge National Laboratory, Oak Ridge, Tennessee, 37831 USA

[5]Department of Material Science and Engineering, College of Engineering, Texas A&M University, College Station, TX 77843, USA.

[6]Center for Remote Health Technologies and Systems, Texas A&M University, College Station, TX 77843, USA.

*Corresponding author E-mail: gaharwar@tamu.edu (AKG)

Keywords: Nanomaterials; Nanosilicates; Protein corona; Hemostatic.

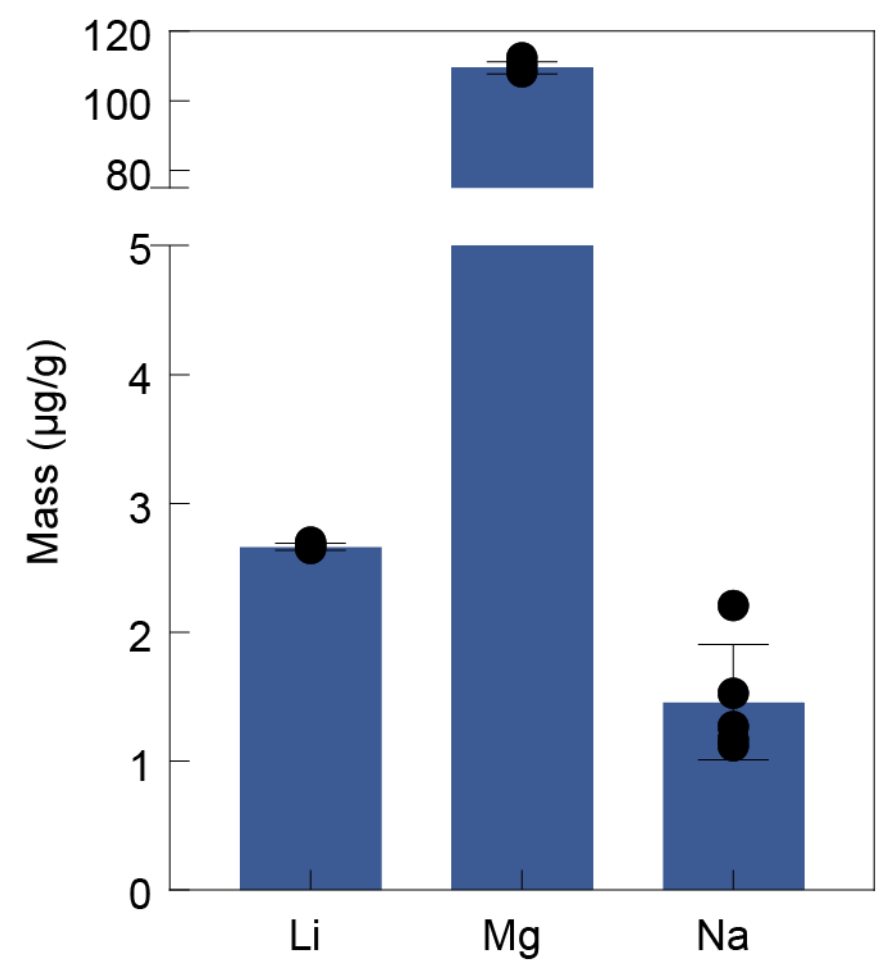

Figure S1: Quantitative elemental mass concentrations (µg/g) of Li, Mg, and Na determined by ICP–MS analysis.

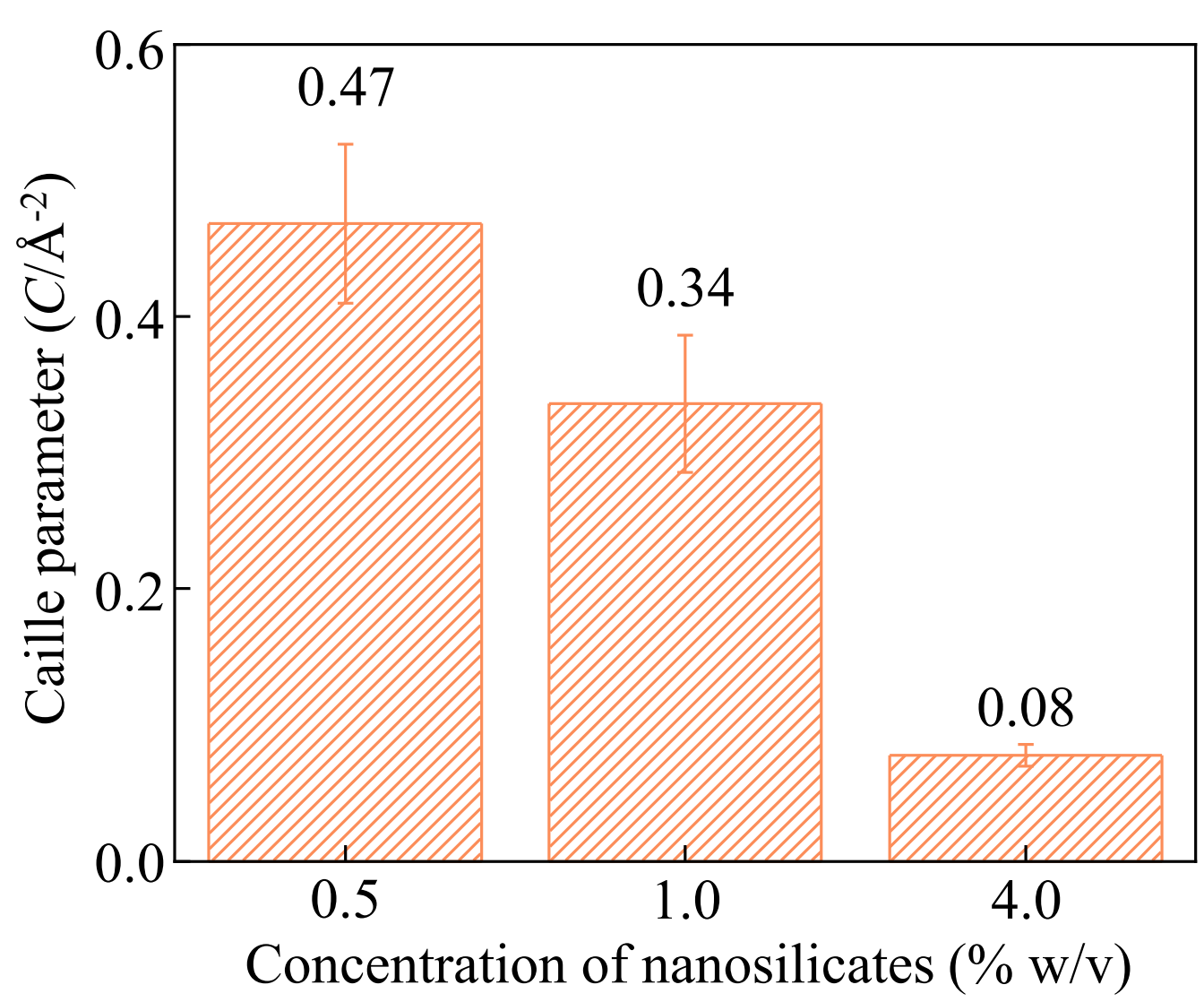

Figure S2. Caille parameter as a function of nanosilicate concentration.

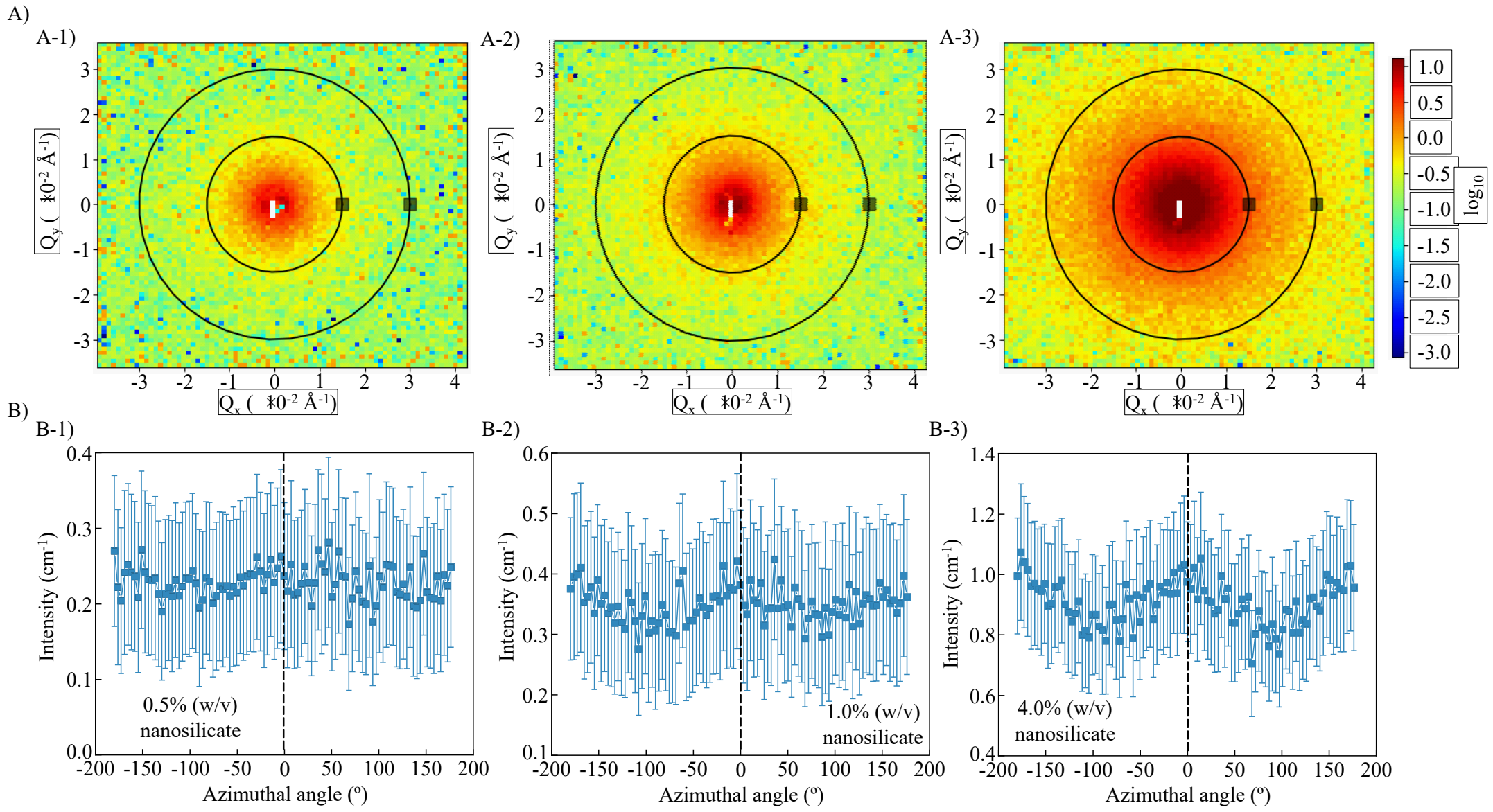

Figure S3. A) 2D scattering patterns and **B)** intensity variations of the nanosilicates at different concentrations.

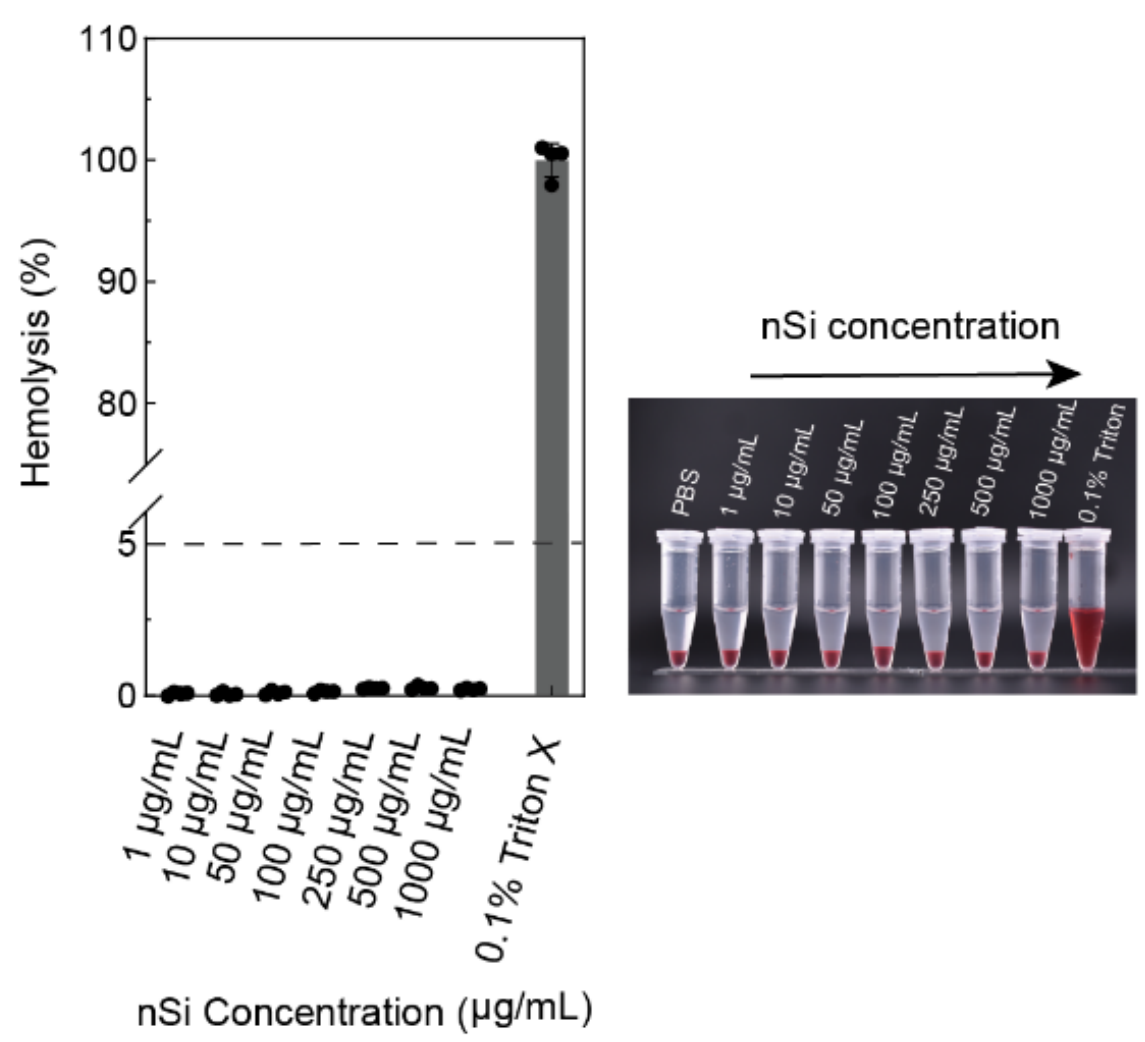

Figure S4. Hemolytic assessment of nSi at various concentrations (1μg/mL-1000μg/mL). The percent hemolysis or hemolytic index is relative to the positive control (0.1% Triton X) and negative control (PBS) and is indicated on the y-axis. The dashed line at 5% hemolysis indicates the generally accepted limit for hemolysis with below 5% is considered non-hemolytic and above considered hemolytic (data ± SD, n=4). Results are represented visually with representative samples.

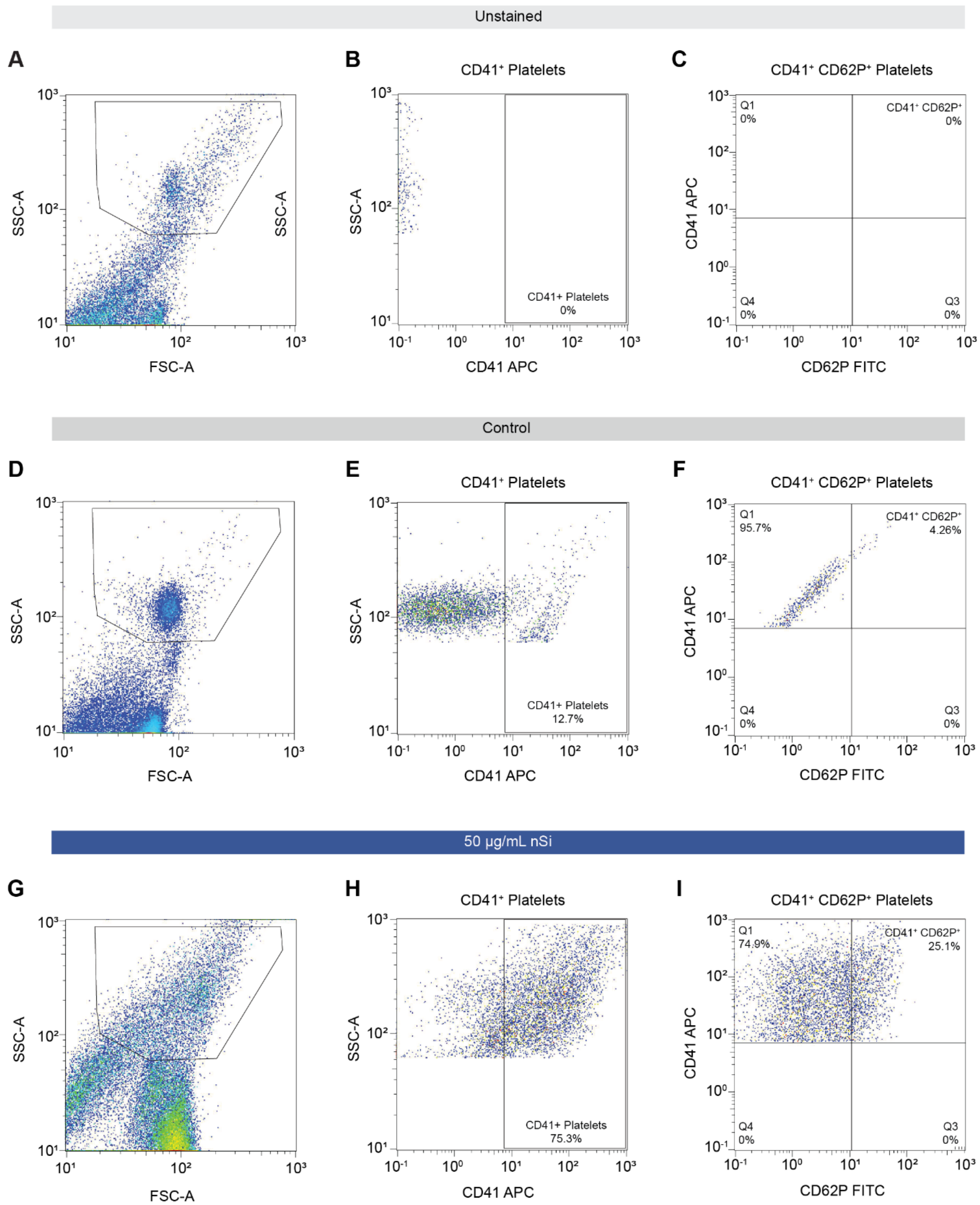

Figure S5: Representative gating strategy used to identify the CD^{41+} platelet population and its activation by nanosilicates. The images representing gating strategy of a control value in Figure S4 (F) and Figure S5 (B) are same. The images representing gating strategy of a 50 µg/mL nSi value in Figure S4 (I) and Figure S5 (E) are same.

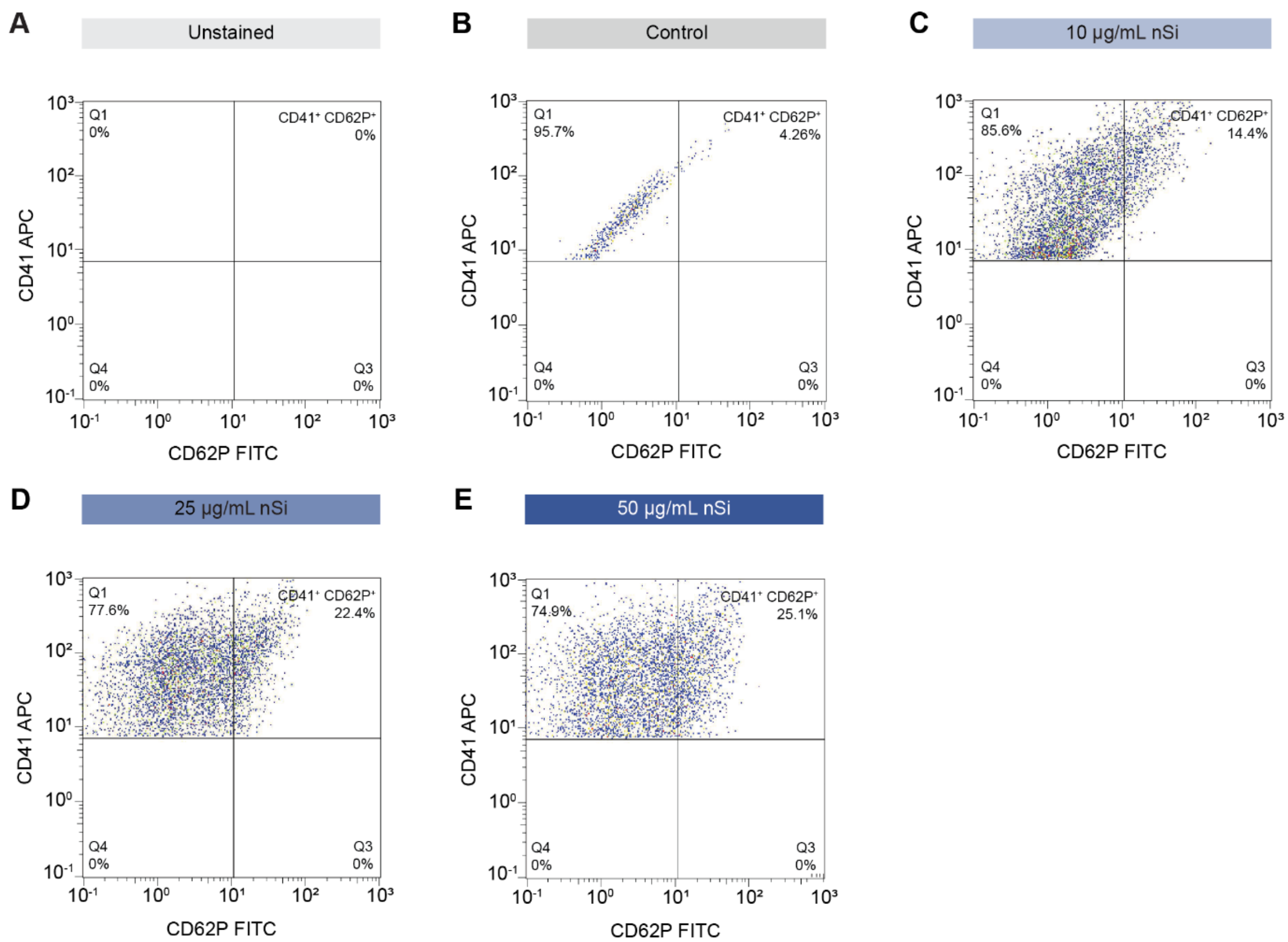

Figure S6: Representative gating strategy used to identify the CD^{41+} - $CD62P^{+}$ platelet population and its activation by nanosilicates. The images representing gating strategy of a control value in Figure S4 (F) and Figure S5 (B) are same. The images representing gating strategy of a 50 µg/mL nSi value in Figure S4 (I) and Figure S5 (E) are same.

Table S1. Parameters for fitting the USANS data of nanosilicates.

Parameters	Values	References	Target parameters
$scale_{particle}$ (0.5%)	0.00189		
$scale_{particle}$ (1.0%)	0.00378	[S1]	
$scale_{particle}$ (4.0%)	0.01512		t, C
ρ_p	$4.18\times10^{-6}/\AA^{-2}$	[S2]	
ρ_s	$6.38\times10^{-6}/\AA^{-2}$		

Table S2. Parameters for fitting the EQ-SANS data of nanosilicates.

Parameters	Values	References	Target parameters
$scale_{particle}$ (0.5%)	0.00189		
$scale_{particle}$ (1.0%)	0.00378	[S1]	
$scale_{particle}$ (4.0%)	0.01512		R, L
ρ_p	$4.18\times10^{-6}/\AA^{-2}$	[S2]	
ρ_s	$6.38\times10^{-6}/\AA^{-2}$		

References

[S1] A. Nelson, T. Cosgrove, Small-angle neutron scattering study of adsorbed Pluronic tri-block copolymers on Laponite. *Langmuir* **2005**, *21(20)*, 9176-9182.

[S2] A. Nelson, T. Cosgrove, A small-angle neutron scattering study of adsorbed poly(ethylene oxide) on Laponite. *Langmuir* **2004**, *20(6)*, 2298-2304.
